# A Systematic Review and Meta-Analysis of 16S rRNA and Cancer Microbiome Atlas Datasets to Characterize Microbiota Signatures in Normal Breast, Mastitis, and Breast Cancer

**DOI:** 10.3390/microorganisms13020467

**Published:** 2025-02-19

**Authors:** Sima Kianpour Rad, Kenny K. L. Yeo, Fangmeinuo Wu, Runhao Li, Saeed Nourmohammadi, Yoko Tomita, Timothy J. Price, Wendy V. Ingman, Amanda R. Townsend, Eric Smith

**Affiliations:** 1 Solid Tumour Group, Basil Hetzel Institute for Translational Health Research, The Queen Elizabeth Hospital, Central Adelaide Local Health Network, Woodville South, Adelaide, SA 5011, Australia; sima.kianpourrad@adelaide.edu.au (S.K.R.); kenny.yeo@adelaide.edu.au (K.K.L.Y.); fangmeinuo.wu@adelaide.edu.au (F.W.); runhao.li@adelaide.edu.au (R.L.); saeed.nourmohammadi@adelaide.edu.au (S.N.); yoko.tomita@sa.gov.au (Y.T.); timothy.price@sa.gov.au (T.J.P.); amanda.townsend@sa.gov.au (A.R.T.); 2 Adelaide Medical School, The University of Adelaide, Adelaide, SA 5005, Australia; wendy.ingman@adelaide.edu.au; 3 Medical Oncology, The Queen Elizabeth Hospital, Central Adelaide Local Health Network, Woodville South, Adelaide, SA 5011, Australia; 4 Robinson Research Institute, The University of Adelaide, Adelaide, SA 5005, Australia; 5 Discipline of Surgery, The University of Adelaide, Adelaide, SA 5005, Australia

**Keywords:** microbiota, microbiome, bacterial community, 16S rRNA, 16S rRNA sequencing, breast cancer, mastitis, human, systematic review, meta-analysis

## Abstract

The breast tissue microbiome has been increasingly recognized as a potential contributor to breast cancer development and progression. However, inconsistencies in microbial composition across studies have hindered the identification of definitive microbial signatures. We conducted a systematic review and meta-analysis of 11 studies using 16S rRNA sequencing to characterize the bacterial microbiome in 1260 fresh breast tissue samples, including normal, mastitis-affected, benign, cancer-adjacent, and cancerous tissues. Studies published until 31 December 2023 were included if they analyzed human breast tissue using Illumina short-read 16S rRNA sequencing with sufficient metadata, while non-human samples, non-breast tissues, non-English articles, and those lacking metadata or using alternative sequencing methods were excluded. We also incorporated microbiome data from The Cancer Genome Atlas breast cancer (TCGA-BRCA) cohort to enhance our analyses. Our meta-analysis identified *Proteobacteria*, *Firmicutes*, *Actinobacteriota*, and *Bacteroidota* as the dominant phyla in breast tissue, with *Staphylococcus* and *Corynebacterium* frequently detected across studies. While microbial diversity was similar between cancer and cancer-adjacent tissues, they both exhibited a lower diversity compared to normal and mastitis-affected tissues. Variability in bacterial genera was observed across primer sets and studies, emphasizing the need for standardized methodologies in microbiome research. An analysis of TCGA-BRCA data confirmed the dominance of *Staphylococcus* and *Corynebacterium*, which was associated with breast cancer proliferation-related gene expression programs. Notably, high *Staphylococcus* abundance was associated with a 4.1-fold increased mortality risk. These findings underscore the potential clinical relevance of the breast microbiome in tumor progression and emphasize the importance of methodological consistency. Future studies to establish causal relationships, elucidate underlying mechanisms, and assess microbiome-targeted interventions are warranted.

## 1. Introduction

The intratumoral microbiome has been extensively studied across various cancers, including breast cancer, where its potential role in tumor pathogenesis and progression has garnered significant interest [1,2]. As the most common malignancy among women worldwide, breast cancer is increasingly recognized as being influenced by the tumor microenvironment, including its associated microbiota [3,4]. Bacteria can modulate cancer progression through various mechanisms, either promoting or inhibiting tumor growth depending on the species involved [5]. For example, *Staphylococcus aureus* has been shown to enhance PD-L1 expression and recruit tumor-infiltrating CD8+ T cells in an in vivo model of triple-negative breast cancer [6]. Conversely, *Fusobacterium nucleatum* binds to sugar moieties on breast cancer cells, a mechanism linked to reduced T-cell infiltration and increased metastatic potential [7]. These findings highlight the need for a deeper understanding of the breast tissue microbiome and its role in cancer biology.

Although breast cancer is generally considered a malignancy with a low bacterial load compared to gastrointestinal cancers, multiple studies have confirmed the presence of microbes in breast tissue. Most of these studies employed 16S ribosomal RNA (rRNA) amplicon sequencing targeting variable regions of the bacterial gene, predominantly using Illumina’s short-read sequencing technology [8,9,10,11,12,13,14,15,16,17,18,19,20,21,22,23,24,25,26,27,28,29,30,31,32,33,34,35,36]. A range of sample types has been analyzed, including healthy, benign, cancerous, and adjacent tissues [8,9,10,11,12,13,14,15,16,17,18,19,20,21,22,23,24,25,26,27,28,29,30,31,32,33,34,35,36]. The potential relationship between the microbiome and breast cancer is often investigated by comparing microbial profiles between cancerous and cancer-adjacent tissues.

The breast microbiome is predominantly composed of bacteria from the phyla *Proteobacteria*, *Firmicutes*, *Actinobacteria*, and *Bacteroidetes*. Frequently reported genera include *Staphylococcus* [8,9,10,11,16,17,20,21,29,36,37,38], *Pseudomonas* [11,12,15,17,20,22,29,33,37,38], *Acinetobacter* [9,11,15,17,21,22,38], *Corynebacterium* [9,10,16,17,36,38], *Propionibacterium* [15,17,20,36], *Prevotella* [17,22,38], *Ralstonia* [9,10,20,22,33], *Streptococcus* [10,11,16,22,31,33], *Burkholderia* [9,10,37,39], and *Methylobacterium* [9,18,36]. Across studies comparing cancerous and cancer-adjacent tissues, *Proteobacteria* [8,9,10,11,15,16,17,20,22,31,33,36,37,38,40] and *Firmicutes* [8,9,10,11,15,16,17,20,31,36,38] are consistently enriched in cancerous tissues. At the genus level, *Staphylococcus* [11,12,16,17,33], *Pseudomonas* [9,15,16,17], *Ralstonia* [12,18,33], *Acinetobacter* [11,22,29], *Corynebacterium*, and *Bacillus* [10,12,17] are frequently elevated in cancer compared to adjacent tissues. However, inconsistencies in taxonomic abundance across studies present challenges in establishing a definitive microbial signature associated with breast cancer.

Several systematic reviews and meta-analyses have attempted to consolidate breast tissue microbiome data [41,42,43]. While some extracted microbial data from published studies [42], others re-analyzed existing datasets [41,43]. However, these meta-analyses have limitations, including the lack of a standardized bioinformatics approach, failure to control for confounding factors (e.g., sequencing depth, inter-patient variability, and primer biases), and inclusion of formalin-fixed paraffin-embedded (FFPE) tissues, which are prone to contamination and degradation artifacts [44]. These inconsistencies may contribute to conflicting findings and hinder a comprehensive understanding of the role of the breast microbiome in cancer.

To address these gaps, we conducted a systematic review and meta-analysis of 11 studies that employed 16S rRNA short-read sequencing to characterize the bacterial microbiome across fresh breast tissue samples, including normal, mastitis-affected, benign, benign-adjacent, cancer-adjacent, and cancerous tissues [8,9,10,11,12,13,15,16,17,19,45]. By exclusively analyzing fresh tissue samples, we aimed to minimize the contamination and degradation issues associated with FFPE samples, ensuring a higher data reliability. To enhance consistency, all the datasets were uniformly re-analyzed and batch-corrected where appropriate, reducing inter-study variability. Additionally, microbial profiles from The Cancer Genome Atlas (TCGA) breast cancer cohort (TCGA-BRCA) [46] were incorporated to complement the 16S rRNA sequencing data, providing further insights into the relationships between the microbiome, clinical features, and tumor phenotypes.

## 2. Materials and Methods

### 2.1. Selection Criteria, Database Search, and Study Design

Our systematic review methodology followed the Preferred Reporting Items for Systematic Reviews and Meta-Analyses (PRISMA) guidelines [47], ensuring a comprehensive and reproducible research process. The systematic review was registered on PROSPERO (Registration number: CRD42024503371).

A comprehensive literature search was conducted in PubMed, Embase, and Scopus using the search terms defined in File S1. The search was restricted to articles published up to 31 December 2023. The titles and abstracts of the identified records were screened by three independent reviewers (S.K.R., S.N., and E.S.). The inclusion criteria were the following: studies that (1) utilized human breast tissue samples, (2) provided sufficient metadata to distinguish different tissue types, (3) used fresh, fresh–frozen, or fresh tissue preserved in nucleic acid stabilizer, and (4) employed Illumina short-read amplicon sequencing of 16S rRNA genes. The exclusion criteria were the following: (1) reviews, systematic reviews, published errata, letters, conferences proceedings, non-English articles, (2) non-human samples, (3) non-breast tissue samples (e.g., breast milk, nipple aspirate fluid, feces, gut, saliva, oral, nasal, nasopharyngeal, respiratory, vaginal, urine, and blood), (4) studies lacking a reported project accession number or sufficient metadata for sample classification, and (5) studies utilizing techniques other than Illumina short-read amplicon sequencing of 16S rRNA genes. The risk of bias was assessed using the RoB 2 tool (ROB2_IRPG_beta_v9) [48] (File S2). A PRISMA checklist for abstracts and reviews is provided (File S3).

### 2.2. The Downloading and Pre-Processing of 16S rRNA Datasets

The raw 16S rRNA sequencing of breast tissues were downloaded from the NCBI Sequence Read Archive (SRA) using SRAtoolkit v3.0.6. Samples were divided into six groups: normal, mastitis-affected, benign, benign-adjacent, cancer, and cancer-adjacent tissues. Cancer tissues were obtained directly from tumors, while cancer-adjacent tissues were from cancer-free regions located within 2 to 5 cm from the tumor. Normal tissues were obtained from either healthy individuals or contralateral (non-cancerous) sites of cancer patients.

Primer removal and sequence denoising were performed using the Cutadapt and Divisive Amplicon Denoising Algorithm 2 (DADA2) plugins in QIIME2 [49,50,51]. Amplicon Sequence Variants (ASVs) classification was performed using the QIIME2 and SILVA reference databases (version silva-138-99-nb-classifier) [49,52]. Batch adjustment was performed using MMUPHin v1.18 (Meta-Analysis Methods with a Uniform Pipeline for Heterogeneity in microbiome studies) in R to adjust for study batch effects (File S4). Initially, we attempted to include all of the samples irrespective of their primer set. However, this approach proved suboptimal due to significant batch effects that remained even after batch correction (File S4). As a result, analyses were conducted separately for each primer set. The V3V4 primer set, being the only one with multiple studies and tissue types, was processed using MMUPHin analysis, and the adjusted values were used for subsequent analyses (refer to File S4). The pre-processing for raw microbial data was described previously [53]. Briefly, ASVs were agglomerated into the genus level since the short-read amplicon sequencing of the 16S rRNA gene is mostly limited to a genus-level resolution [53,54]. Low-abundance ASVs were filtered using PreFL from PLSDA-batch v1.0.0 with the parameters keep.spl = 10 and keep.var = 0.01 [55].

### 2.3. Alpha and Beta Diversity Analyses

Both conventional (rarefied) and compositional (central log ratio transformed) methods were applied to analyze alpha and beta diversities, as previously described [56,57,58]. For the conventional dataset, samples were rarefied to an even depth (read = 1000) using rarefy_even_depth in phyloseq v1.48 [59]. For alpha diversity, the rarefied or unrarefied abundance Shannon index was used to measure alpha diversity using microeco v1.9.1 R package [60]. Differences in alpha diversities between tissue types (cancer, cancer-adjacent, normal, and mastitis tissue) were tested using the Kruskal–Wallis test with Dunn’s multiple comparison and the Mann–Whitney test. For beta diversity analysis, rarefied relative abundances of all genera were ordinated using Bray–Curtis distances and visualized with Principal Coordinates Analysis (PCoA) utilizing the phyloseq v1.46 and ggpubr v0.6 R packages [59]. A permutational multivariate analysis of variance (PERMANOVA) was used to test differences in the beta diversity between tissue types, while the dispersion (variance) among different tissue types was evaluated using the betadisper test from the vegan v2.6 package [61]. Since PERMANOVA relies on the assumption of the homogeneity of dispersion among groups, the betadisper test was used to detect potential differences in dispersion, as a significant variation in dispersion could impact the validity of PERMANOVA results. All the tests were performed with 999 permutations. To address the compositional nature of microbiome data, a CLR transformation with an offset of 0.5 was applied to generate scale-invariant values, mitigating biases caused by differences in library sizes [56,62]. The CLR-transformed abundance data were subsequently analyzed using Euclidean distances and visualized with PCoA.

### 2.4. The Differential Abundance and Prevalence Between Different Breast Tissues

The mean relative abundance and prevalence were calculated at the phylum and genus levels using phyloseq v1.48 [59]. The general breast microbiome composition and prevalence at these taxonomic levels were assessed across all the breast tissue samples for each primer set. To investigate differences in the relative abundance and CLR-abundance between cancer and cancer-adjacent samples, statistical tests were performed at the genus level. Multiple Mann–Whitney tests with Bonferroni correction for multiple comparisons were used for unpaired cancer and cancer-adjacent samples, while multiple Wilcoxon tests with Bonferroni correction were applied for paired samples. All statistical analyses were conducted using Prism 10 and mean relative abundance plots were visualized using the microeco R package, excluding samples with zero reads.

### 2.5. The Re-Analysis of Breast Tissue Microbiome Data from the Cancer Genome Atlas

The microbiome dataset for The Cancer Genome Atlas Breast Cancer (TCGA-BRCA) cohort was obtained from Sepich-Poore et al. (2024) [63]. Specifically, we used the “RS210-clean” dataset, which comprises host-depleted microbiome reads with ≥50% aggregate coverage [63]. This dataset includes microbiome profiles from 112 cancer tissues and 16 cancer-adjacent tissues, including 15 paired cancer and cancer-adjacent samples. Microbial data were aggregated at the genus level for consistency with the 16S rRNA dataset analysis. It is important to note that the TCGA-BRCA samples were not collected under sterile conditions, as the cohort was not initially designed for microbiome research. To address this, we excluded likely contaminants using two criteria: (1) taxa that are not present in the 16S rRNA datasets, and (2) taxa present but at an abnormally high abundance. After excluding these contaminants, microbial reads were normalized relative to the total BAM-mapped reads and scaled to 10^9^ reads to account for differences in sequencing depth [64]. All analyses for the TCGA-BRCA dataset followed the same workflow as for the 16S rRNA sequencing data. An exception was applied for CLR-abundance calculations, where an offset of 0.01 was used.

### 2.6. Microbial Correlation Analysis with Tumor Phenotype and Clinical Survival Data

The tumor tissue phenotype was characterized using the TCGA-BRCA transcriptomics dataset, as previously described [65]. A total of 29 functional gene expression signature (FGES) scores were utilized to represent the major functional characteristics, as well as immune, stromal, and other cellular populations, within breast cancer tissues [65]. To assess relationships between microbial CLR-abundance and FGES scores, Spearman’s correlation analysis was performed using the cor.test function in R. *p*-values were adjusted for false discovery rate (FDR) to account for multiple comparisons. Additionally, univariate and Cox proportional hazard model analyses were conducted using the survminer package in R. These analyses evaluated associations between microbial abundance and the overall survival for continuous CLR-abundance data and for high and low CLR-abundance groups, characterized by the top and bottom 30% of CLR-abundance values.

### 2.7. Statistical Analysis

For comparisons made between unpaired tissue groups, the Kruskal–Wallis test with Bonferroni’s multiple comparison was used. For all of the paired cancer and cancer-adjacent samples, the Wilcoxon matched-pairs signed rank test was performed. Statistical analysis was performed using Prism 10 for macOS (Version 10.4.0 (527), 23 October 2024; GraphPad Software Inc., La Jolla, CA, USA).

## 3. Results

### 3.1. Study Selection

**Figure 1:**
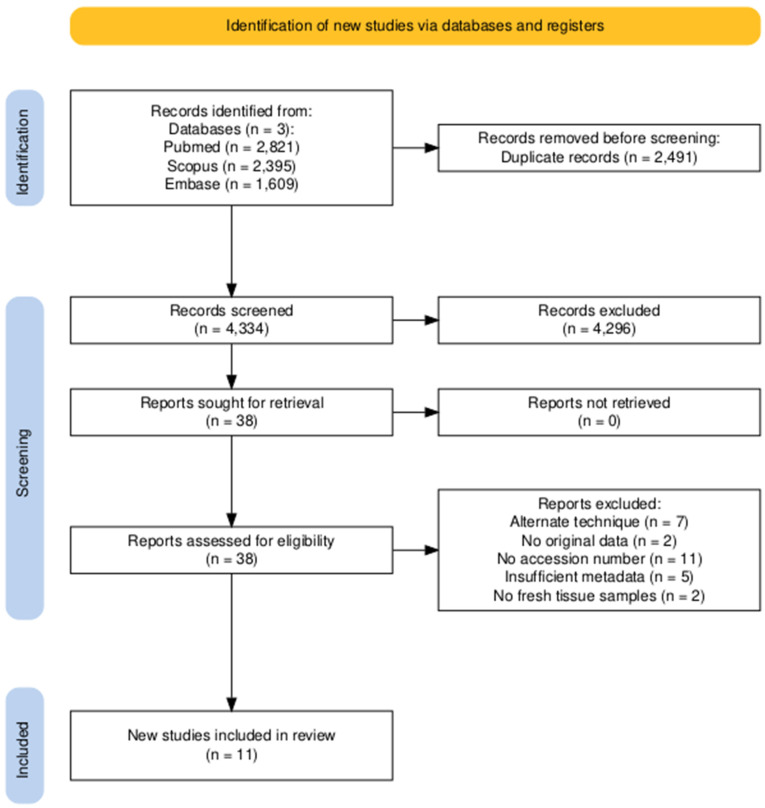
Flow diagram illustrating the study identification, screening, and selection process, conducted in accordance with the Preferred Reporting Items for Systematic Reviews and Meta-Analyses (PRISMA) guidelines [47].

A comprehensive search of PubMed, Scopus, and Embase databases identified 6825 records published up to 31 December 2023 (Figure 1). After removing duplicates, 4334 records remained for screening. Title and abstract screening excluded 4296 records based on irrelevance, including studies unrelated to the microbiome, non-human microbiome studies and microbiome studies of non-breast tissue samples including breast milk, nipple aspirate fluid, feces, gut, saliva, oral, nasal, nasopharyngeal, respiratory, vaginal, urine, and blood. This left 38 full-text articles for eligibility assessment.

Of these, 27 were excluded based on predefined criteria (Table S1). Seven did not utilize 16S rRNA short-read sequencing [37,38,66,67,68,69,70], and two re-used previously reported datasets without presenting original data [41,43]. Eleven lacked project accession numbers [14,18,21,23,24,26,27,32,33,34,36], while five were excluded due to insufficient metadata [20,25,28,31,35]. Despite attempts to contact the corresponding authors, the required project accession numbers or metadata remained unavailable. Additionally, two studies were omitted for using only FFPE tissue rather than fresh samples [22,29].

After applying these criteria, 11 studies met the eligibility requirements and were included in the meta-analysis [8,9,10,11,12,13,15,16,17,19,45]. Collectively, these studies provided 16S rRNA sequencing data for 1260 fresh, fresh–frozen, or nucleic acid-preserved breast tissue samples, including 532 normal, 269 cancerous, 372 cancer-adjacent, 15 benign, 24 benign-adjacent, and 48 mastitis-affected samples (Table 1). All the studies used an Illumina sequencing platform. A risk of bias assessment using the RoB2 tool indicated an overall low risk of bias across all 11 included studies. All of the studies contained the required metadata and 16 rRNA sequencing data, and patients were treated similarly according to the inclusion and exclusion criteria (File S2).

Two studies employed multiple 16S rRNA primer sets. Nejman et al. used V2, V3, V5, V6, and V8 [16], while German et al. used V1V2, V2V3, V3V4, V4V5, V5V7, and V7V9 [9]. To ensure consistency and comparability across the studies, we utilized only the V6 primer data from Nejman et al. and the V3V4 primer data from German et al., as these regions were also used in other studies included in this meta-analysis. Analyses of the excluded primer sets were comprehensively reported in their respective publications [9,16].

### 3.2. The General Composition of the Breast Microbiome at the Phylum and Genus Level

**Figure 2:**
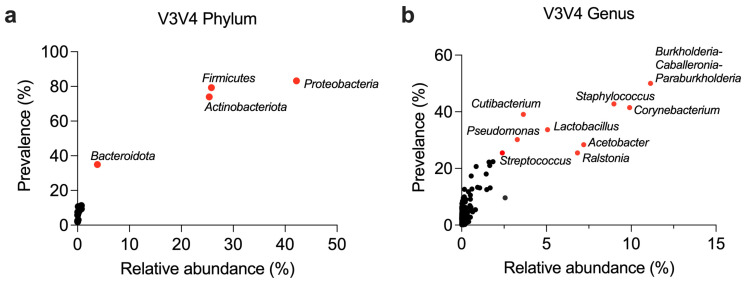
General composition of the breast microbiome in 16S rRNA V3V4-sequenced samples. The prevalence and mean relative abundance of taxa are shown at the (**a**) phylum and (**b**) genus levels for the V3V4 primer set (unrarefied, *n* = 832). The top phyla and genera are labelled and highlighted with red symbols to indicate prominence in the dataset.

Despite extensive research on the breast microbiome, inconsistencies persist in defining its core composition. To address this, we analyzed microbiome profiles from 832 breast tissue samples, including 509 normal, 154 cancer-adjacent, 121 cancerous, and 48 mastitis-affected tissues from five studies employing V3V4 primers [9,10,11,12,13] (Figure 2, Table 2) and additional studies employing other primer sets [8,14,15,16,17,19] (Figures S1–S4, Tables S2 and S3).

An analysis of the pooled V3V4 datasets revealed that *Proteobacteria*, *Firmicutes*, *Actinobacteriota*, and *Bacteroidata* were the most abundant (mean relative abundance ≥ 3.8%) and prevalent (≥35%) phyla across the normal, mastitis-affected, cancer-adjacent, and cancerous breast tissues included in these studies (Figure 2a, Table 2 and Table S2). These phyla were consistently among the most abundant and most detected (prevalence ≥ 41%) in datasets generated with other primer sets (V1V3, V3V5, V4, V4V6, and V6) (Figure S1, Table S2). An exception was observed with *Campilobacterota*, which ranked as the third most abundant phylum in the V1V3 primer dataset (abundance 3.4%, prevalence 12%). These findings remained robust even after rarefying the datasets to standardize the sequencing depth, ensuring reliable comparisons (Figure S2). Collectively, these results indicate that the breast microbiome predominantly consists of *Proteobacteria*, *Firmicutes*, *Actinobacteriota*, and *Bacteroidata*, regardless of the primer set or study.

Given the limitations of short-read 16S rRNA sequencing in resolving taxa beyond the genus level [53], we further analyzed the datasets at the genus level. Pooled V3V4 primer datasets identified 303 bacterial genera, of which 13 exhibited a prevalence > 20% across all breast samples (Table S2). Among these, *Burkholderia-Caballeronia-Paraburkholderia*, *Corynebacterium*, *Staphylococcus*, *Acetobacter*, *Ralstonia*, and *Lactobacillus* were the most abundant, each with an abundance > 5% and a prevalence > 25% (Table 2).

*Staphylococcus* emerged as one of the most prevalent genera, identified in 42.8% of the breast tissue samples, with a mean relative abundance of 9.0% in the pooled V3V4 dataset (Figure 2b, Table 2). Detection rates varied by primer set, ranging from 19% with V1V3 primers to 100% with V3V5 or V4 primers (Figures S3 and S4, Tables S2 and S3). Its relative abundance also showed substantial variation, from 0.28% (V1V3 primers) to 18.8% (V3V5 primers). Other genera with >20% prevalence in the pooled V3V4 dataset included *Cutibacterium* (mean relative abundance: 3.6%), *Pseudomonas* (3.3%), *Streptococcus* (2.4%), *Sphingomonas* (1.8%), *Bacillus* (1.7%), *Acinetobacter* (1.6%), and *Paracoccus* (0.9%). These genera were consistently detected across primer sets, apart from *Bacillus*, which was absent from the dataset V3V5 dataset (Tables S2 and S3).

Significant discrepancies in bacterial genera detection were observed between studies, reflecting the influence of primer selection and study design. For example, *Brochothrix*, a bacterium commonly associated with food spoilage, was highly abundant (mean relative abundance 30.4%) and prevalent (64%) in metastatic cancer samples from lymph nodes [8]. In contrast, it was detected at substantially lower levels in the pooled V3V4 (abundance 0.01%, prevalence 0.5%) and V3V5 (0.02%, 3.6%) datasets and was undetected with other primer sets (Table S2). Similarly, *Psychrobacter,* typically found in environments with moderate to high salinity and low temperatures [71], was frequently identified with V1V3 (prevalence 58.7%, abundance 8.2%) and V4 (87.2%, 1.3%) primers. In contrast, it was detected at a much lower abundance and prevalence with V3V4 (0.3%, 0.4%) and V4V6 (7.4%, 2.9%) primer sets (Table S2). *Halomonas*, a genus commonly associated with saline and hypersaline environments, showed a high prevalence and abundance in breast samples analyzed using the pooled V6 (prevalence 59.5%, abundance 43.9%) and V4 (prevalence 62.8%, abundance 0.9%) primers. In contrast, it was detected at significantly lower levels with the pooled V3V4 primers (prevalence 0.6%, abundance 0.03%) and was undetectable with other primer sets (Tables S2 and S4). Notably, a high prevalence (100%) and abundance (75.3%) were observed in a single study using the V6 primer set [16] (Table S4), while other studies reported a considerably lower prevalence (<3.1%), except for the V4 primer set [19] and abundance (<0.1%) (Tables S2 and S4). These trends remained consistent following rarefaction (Tables S3 and S5). These findings underscore the significant influence of primer selection and study design on the observed prevalence and abundance of bacterial genera in the breast microbiome.

Collectively, these results indicate that the breast microbiome is predominantly composed of *Proteobacteria*, *Firmicutes*, *Actinobacteriota*, and *Bacteroidota*, with *Staphylococcus* frequently detected, regardless of study or primer set. However, the prevalence and abundance of specific bacterial genera vary across breast tissue types, primer sets, and study designs. This variability underscores the potential existence of distinct microbial signatures associated with different breast tissue conditions (normal, cancer, cancer-adjacent, and mastitis) and highlights the need for standardization in future microbiome research.

### 3.3. Similar Microbial Diversity Between Cancer and Cancer-Adjacent Breast Tissue, but Distinct from Mastitis and Normal Tissues

**Figure 3:**
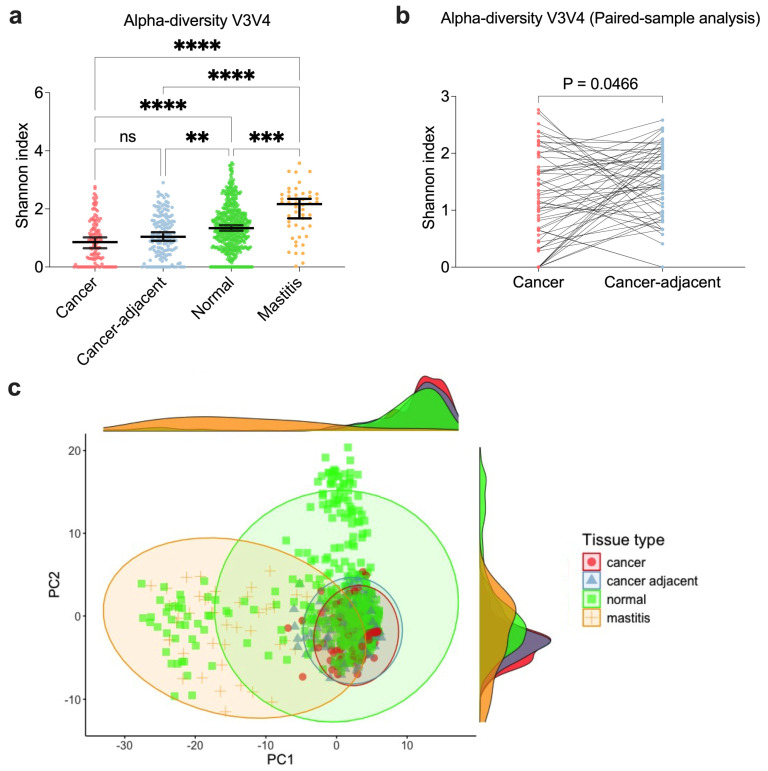
Alpha and beta diversities for V3V4 breast tissue samples. (**a**) Shannon index representing alpha diversity across cancer, cancer-adjacent, normal, and mastitis-effected breast tissue samples. (**b**) Shannon index for paired cancer and cancer-adjacent samples. (**c**) PCoA density plot based on the Euclidean distance of CLR-transformed abundance, illustrating beta diversity. The Kruskal-Wallis test with Dunn’s multiple comparison was performed for unpaired analysis in (**a**), while the Wilcoxon paired signed-rank test was performed for paired sample analyses in (**b**). **** *p* < 0.0001, *** *p* < 0.001, ** *p* < 0.01, not significant (ns), *p* > 0.05.

To further characterize the microbial composition at the genus level across various breast tissue types, we focused on cancer and cancer-adjacent tissues. Using pooled V3V4 primer set data, which includes the broadest range of tissue types (cancer, cancer-adjacent, mastitis, and normal), we compared the microbial profiles of cancer tissues with those of other breast tissue types (Figure 3).

Alpha diversity, measured by the Shannon index, showed no significant differences between cancer tissues (median = 0.86, 95% CI = 0.77–1.04) and cancer-adjacent tissues (median = 1.04, 95% CI = 0.97–1.21) (Figure 3a). A minimal but statistically significant difference (median difference −0.36, 95% CI = −0.50–0.0004; *p* = 0.0466) was observed between paired samples from cancer and cancer-adjacent tissues (Figure 3b). These results were consistent across datasets generated using other primer sets (V1V3 and V4V6) that included paired cancer and cancer-adjacent tissues (Figure S5). In contrast, alpha diversity was significantly higher in mastitis-affected (median = 2.17, 95% CI = 1.66–2.16) and normal tissues (median = 1.34, 95% CI = 1.30–1.44) relative to cancer and cancer-adjacent tissues (Figure 3a). This trend remained consistent in the rarefied dataset analyses (Figure S6).

Beta diversity, representing the overall microbial composition, was evaluated using PCoA plots of Euclidean distances on CLR-transformed abundance data (Figure 3c and Figure S7), PCoA density plots based on Bray–Curtis distances from rarefied datasets (Figure S8), and PERMANOVA tests. Slight differences in beta diversity were observed between cancer and cancer-adjacent tissues for datasets using V3V4 (PERMANOVA: R^2^ = 0.005, *p* = 0.028) and V4V6 (PERMANOVA: R^2^ = 0.034, *p* = 0.002) primers (Table S6). However, no significant differences were found between these tissue types in the V1V3 dataset (Table S6). Greater differences in beta diversity were noted in paired cancer and cancer-adjacent samples (PERMANOVA: V1V3: R^2^ = 0.015, *p* = 0.03; V3V4: R^2^ = 0.012, *p* = 0.004; V4V6: R^2^ = 0.03, *p* = 0.028) (Table S8). Mastitis-effected and normal tissues showed significant differences in beta diversity compared to cancer tissues (PERMANOVA: *p* < 0.05). However, the Betadisper analysis revealed that these differences were primarily driven by greater variability within each group rather than consistent differences in their central microbial composition (*p* < 0.05). This suggests that the microbial communities in these groups exhibited a greater spread or variability rather than distinct central compositions. These trends were consistent across analyses using Bray–Curtis distances from rarefied datasets (Table S7).

In summary, cancer and cancer-adjacent tissues exhibit similar microbial diversity, while mastitis-affected and normal tissues show significantly higher alpha diversity and distinct beta diversity. These differences likely reflect underlying variations in tissue conditions and microbial environments.

### 3.4. Similar Genus-Level Abundance Between Cancer and Cancer-Adjacent Breast Tissue Samples

We examined genus-level abundance differences between cancer and cancer-adjacent tissues. *Staphylococcus* and *Corynebacterium* were among the most abundant bacteria identified in the cancer and cancer-adjacent tissues using the V3V4 primers. Consistent with beta-diversity findings, no significant changes were observed in CLR-abundance or relative abundance (both rarefied and non-rarefied) for the V1V3 and V3V4 primer sets (Tables S9 and S10). This was true for both paired and unpaired differential abundance analyses. For the V4V6 primer set, three bacterial genera showed significant differences across various analyses (Table S11). Specifically, *Pseudomonas* was elevated in cancer tissues (paired CLR-abundance analysis, Table S11B), while *Aliterella* and *Rubrobacter* were more abundant in cancer-adjacent tissues. *Aliterella* was significant in paired CLR-abundance (Table S11B) and unpaired non-rarefied relative abundance analyses (Table S11C). In contrast, *Rubrobacter* showed significance in paired rarefied relative abundance analyses (Table S11F). Notably, *Aliterella* was absent in cancer tissues (abundance = 0%) and was only detected in 11 out of 68 samples (16.1% prevalence) for the V4V6 primer set (Tables S2 and S11). Overall, cancer and cancer-adjacent breast tissues exhibited highly similar microbial profiles, with minimal differences observed across all 16S rRNA primer sets, indicating a consistency in microbial composition between these tissue types.

### 3.5. The Microbial Profiles of Cancer and Cancer-Adjacent Breast Tissues in TCGA-BRCA Are Consistent with 16S rRNA Sequencing

Recent seminal studies have shown the presence of microbial reads within TCGA metagenomic datasets [63] offering the opportunity to differentiate between cancer and cancer-adjacent tissue samples and explore correlations between microbial profiles and clinical outcomes. To utilize this dataset, we first investigate whether microbial abundance patterns identified in TCGA-BRCA align with those observed in 16S rRNA sequencing.

The initial analysis revealed that Shigella, Escherichia, Afipia, Staphylococcus, Acinetobacter, Achromobacter, Corynebacterium, Streptococcus, Cutibacterium, and Cupriavidus were the top 10 genera identified in breast samples (Table S12). Notably, Escherichia (mean relative abundance = 32.8%, prevalence = 85.2%) and Shigella (mean relative abundance = 42.4%, prevalence = 85.2%) were unexpectedly dominant (Figure S9, Tables S12 and S13). These genera, which are not typically abundant in breast tissues [72,73], were also absent or minimally represented in our analyses of 16S rRNA datasets (mean relative abundance: V1V3 = not detected (ND) V3V4 = 0.55%, V3V5 = 1.78%, V4V6 = 0.21%, V4 = 2.12%, V6 = ND) (Table S13B). Their elevated presence in TCGA-BRCA may reflect contamination during the sample collection or sequencing [74,75,76]. Similarly, Achromobacter showed an unusually high abundance and prevalence compared to 16S rRNA data (Figure S9, Tables S12 and S13B). In addition, there were 28 bacterial genera that were not found in any 16S rRNA sequencing (Table S13A). As a result, these genera were excluded from subsequent analyses, leaving a total of 88 genera for the TCGA-BRCA dataset (Table S13).

**Figure 4:**
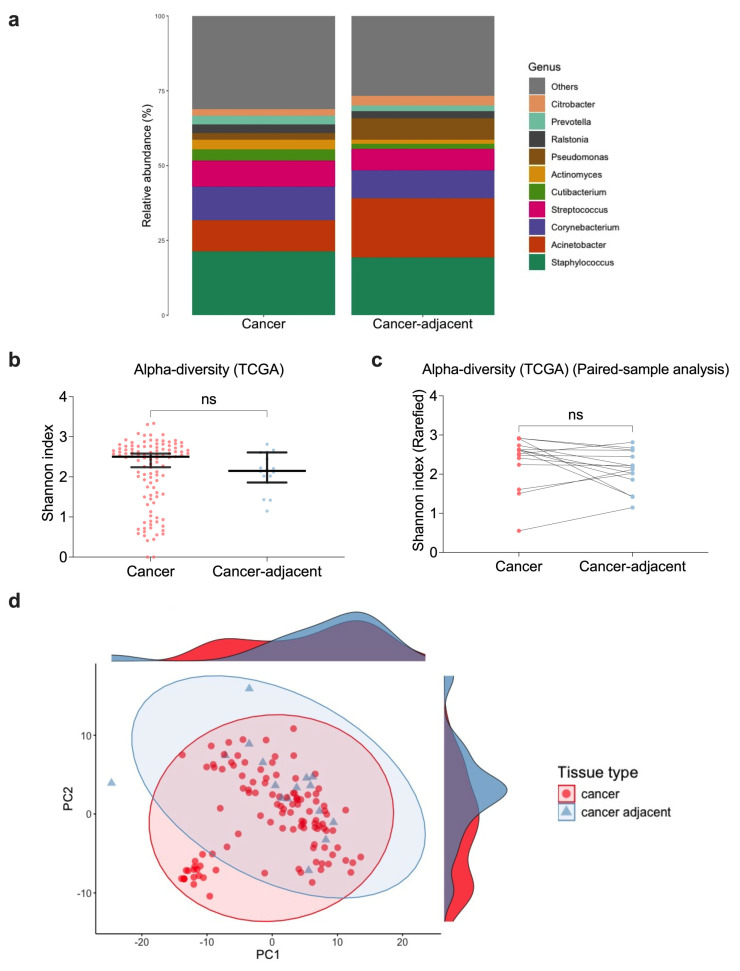
TCGA-BRCA microbial abundance and diversity. (**a**) The mean relative abundance of genera in cancer and cancer-adjacent breast tissue. (**b**) Shannon index for unpaired samples. (**c**) Shannon index for paired cancer and cancer-adjacent samples. (**d**) PCoA density plot based on the Euclidean distances of CLR-transformed abundance data representing beta diversity. The Kruskal-Wallis test with Dunn’s multiple comparison was performed for unpaired analysis (**a**), and the Wilcoxon paired signed-rank test was performed for paired sample analyses (**b**). Not significant (ns), *p* > 0.05.

After filtering, three breast cancer tissue samples were left with no microbial reads. The remaining microbial genera closely resembled those reported in the 16S rRNA sequencing datasets (Figure 4a, Tables S2 and S14). The most prominent genera (>5% mean relative abundance and >80% prevalence) were *Staphylococcus*, *Acinetobacter*, *Corynebacterium*, and *Streptococcus* (Figure 4a and Figure S10 and Table S14). Among these, *Staphylococcus* was the most dominant, with a mean relative abundance of 21.3% (prevalence 88.1%) in cancer tissues and 19.2% (prevalence 100%) in cancer-adjacent tissues (Figure 4a and Figure S10, Table S14). Consistent with 16S rRNA results, no significant differences in alpha diversity (Figure 4b,c) were observed between cancer and cancer-adjacent tissues, while slight differences in beta diversity (PERMANOVA (unpaired): R^2^ = 0.018, *p* = 0.005, beta-dispersion *p* = 0.02. PERMANOVA (paired): R^2^ = 0.035, *p* = 0.052, beta-dispersion *p* = 0.11) were detected (Figure 4d, Table S15). When comparing differences in the genera relative abundance and CLR-abundance, cancer and cancer-adjacent breast tissues exhibited largely similar microbial profiles. Notably, only *Acinetobacter* and *Propionibacterium* were found to differ significantly in unpaired CLR-abundance analyses, while no significant differences were observed in other analyses (Table S16). Overall, the microbiome composition at the genus level appeared to be comparable between cancer and cancer-adjacent breast tissues, like the findings in 16S rRNA sequencing.

### 3.6. The Evaluation of Microbial Abundance with Tumor Phenotype and Overall Survival

Since cancer and cancer-adjacent tissues exhibited similar microbial profiles, we investigated the relationship between prominent microbial genera (*Staphylococcus*, *Acinetobacter*, *Corynebacterium*, and *Streptococcus*) and breast tumor phenotype, as well as patient overall survival. These genera were consistently identified across all 16S rRNA datasets.

**Figure 5:**
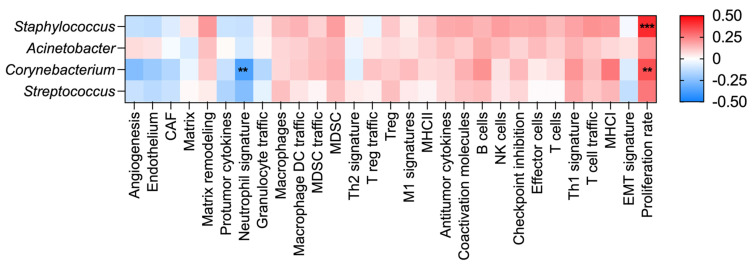
A correlation analysis of the CLR-abundance for prominent bacterial genera in breast cancer tissues. A Spearman correlation analysis was performed to evaluate the relationship between the CLR-abundance of bacterial genera and tumor phenotypes described by 29 functional gene expression signatures (FGES). P-values were adjusted for the false-discovery rate (FDR). *** *p* < 0.001, ** *p* < 0.01.

The tumor phenotypes were characterized using 29 functional gene expression signatures (FGES) that represent key functional characteristics of the cancer tissues [65]. A correlation analysis of CLR-abundance with FGES scores revealed significant associations (Figure 5). Proliferation-related FGES positively correlated with the abundance of *Staphylococcus* (r = 0.42, FDR *p* = 0.000743) and *Corynebacterium* (r = 0.35, FDR *p* = 0.0094). Meanwhile, *Corynebacterium* abundance negatively correlated with the neutrophil-related signature (r = −0.37, FDR *p* = 0.0043).

To evaluate associations between microbial abundance and overall survival, univariate Cox proportional hazards models were performed using both continuous CLR-abundance values and categorized groups. In the categorized analysis, “high” abundance was defined as the top 30% and “low” as the bottom 30% based on the median (Table 3).

Using CLR-abundance as a continuous variable, *Staphylococcus* was identified as the only prominent genus significantly associated with a reduced overall survival (HR = 1.40, 95% CI = 1.04–1.88, *p* = 0.02). Patients with a high *Staphylococcus* abundance demonstrated a 4.1-fold increase in the risk of death compared to those with a low abundance (HR = 4.06, 95% CI = 1.13–14.6, *p* = 0.03) (Table 3).

For *Corynebacterium*, the continuous variable was not associated with a reduced overall survival. However, when patients were categorized into high and low abundance groups, a significant association was observed (HR = 4.72, 95% CI = 1.02–21.9, *p* = 0.048). No significant associations with overall survival were observed for *Streptococcus* and *Acinetobacter* abundance.

## 4. Discussion

The breast microbiome has garnered significant attention in recent years for its potential role in influencing disease phenotypes [6,8,9,10,11,12,13,14,15,16,17,18,19]. While studies employing 16S rRNA sequencing have provided valuable insights into microbial composition in a variety of breast tissue types, including normal, cancer, and cancer-adjacent, findings have been inconsistent [11,15,37]. For instance, whilst the majority of studies to date have suggested that there is little or no difference in the abundance of different bacterial genera between cancer and cancer-adjacent tissues [8,9,12,16,18,20,33,36], some studies have suggested that *Rothia* [11] and *Corynebacterium* [37] are elevated in cancer compared to cancer-adjacent tissues, whilst *Propionibacterium* [15], *Aeromonas* [15], and *Pseudomonas* [11] were less abundant in cancer tissues, highlighting key microbes that may play a clinical role in breast cancer.

Similar to previous studies, we identified *Proteobacteria* [8,11,12,14,15,16,17,18,19,20,31,33,37,38], *Firmicutes* [8,10,11,12,14,16,17,18,19,20,31,33,37,38], *Actinobacteriota* [8,10,11,12,14,15,16,17,18,20,31,33,37,38,39], and *Bacteroidota* [8,12,14,17,18,20,31,33,37,38] as major phyla found within breast tissues, regardless of the disease phenotype or primer sets used. Importantly, these phyla can also be detected using culture-based methods across various samples types, including breast milk [77,78], alveolar skin [78], and breast tissue samples from individuals with diverse phenotypes, such as cancer, cancer-adjacent, benign, benign-adjacent, and inflammatory diseases [78,79]. Growing evidence suggests that these microbial communities may influence breast cancer development and progression through their effects on inflammation, immune modulation, and tumor microenvironment alterations. However, the contributions of these bacteria to breast cancer remain an area of active investigation.

Proteobacteria, the most abundant phylum in breast cancer tissue, appears to play a pivotal role in tumor progression. For instance, Escherichia coli has been shown to alter cancer cell metabolism in vitro in study [80] and induce macrophage redistribution, leading to granulation tissue formation, increased TNFα expression, and enhanced matrix metalloproteinase 9 (MMP9) activity [81], all of which contribute to tumor progression. Additionally, Klebsiella pneumoniae has been implicated in TLR4-mediated inflammation due to its LPS, a pathway that promotes tumor growth, particularly in estrogen receptor-positive (ER+) breast cancer cells (MCF-7) [82]. These findings highlight the potential pro-tumorigenic effects of Gram-negative Proteobacteria, although further studies are needed to determine their precise role in breast cancer progression.

Firmicutes and Bacteroidota are two other phyla with high abundance in breast tissue. Interestingly, breast cancer patients exhibit a predominance of anaerobes, irrespective of whether they belong to Firmicutes or Bacteroidota. A lower abundance of Firmicutes has been associated with decreased low-density lipoprotein (LDL) cholesterol levels, which may indicate metabolic shifts in cancer patients. Additionally, certain Firmicutes members, such as Faecalibacterium spp., correlate with breast cancer stage, with higher abundances observed in advanced stages. These findings suggest that the Firmicutes composition may influence tumor progression through metabolic regulation, though its precise mechanistic role remains unclear [83].

Bacteroidota, including Bacteroides and Prevotella, may influence breast cancer progression through immune regulation and metabolic alterations. A lower Firmicutes-to-Bacteroidetes (F/B) ratio has been associated with BC risk and prognosis, with patients having a family history of breast cancer displaying a significantly lower F/B ratio. Additionally, the serum F/B ratio reflects microbial dysbiosis, which may contribute to BC development through extracellular vesicle (EV)-mediated signaling [83]. These findings suggest that microbial imbalances, rather than the mere presence of specific bacteria, may be a crucial factor in breast cancer pathogenesis.

Actinobacteriota, particularly Corynebacterium and Cutibacterium acnes, has also been identified in breast cancer tissue, but higher in healthy cancer adjacent tissue, with conflicting evidence regarding its role. Some studies suggest that Actinobacteria-derived bioactive metabolites may have anticancer properties, while others indicate that specific species, such as Acinetobacter, may promote tumor progression through inflammation and immune modulation [20]. Some Actinobacteria, such as *Agrococcus* sp., have been linked to increased malignancy, making it challenging to classify this phylum as purely beneficial or harmful [84]. Conversely, Acinetobacter baumannii has been linked to the inhibition of breast tumor invasion, potentially via its glutaminase activity, which influences cancer cell metabolism [85] or by the production of natural anticancer compounds, including Cinerubin B and Actinomycin V, demonstrated antiproliferative effects, inhibition of epithelial–mesenchymal transition (EMT), and suppression of tumor cell migration and invasion [84]. The dual nature of Actinobacteria underscores the complexity of the breast microbiome and its potential role in both tumorigenesis and therapy.

In agreement with previous investigations [9,18,33,38], *Staphylococcus* was the most abundant and prevalent genera, consistently identified across all breast tissues, regardless of the study or primer set used. Other genera, such as *Cutibacterium*, *Pseudomonas*, *Streptococcus*, *Sphingomonas*, *Acinetobacter*, and *Paracoccus* were also detected at a higher prevalence in breast tissues in our meta-analysis. Among these, only *Pseudomonas* [12,15,17,18,20,33,38] and *Acinetobacter* [15,33,38] were also identified among the top abundant genera in previous studies using breast tissues. Importantly, *Staphylococcus*, *Cutibacterium*, *Pseudomonas*, *Streptococcus*, and *Acinetobacter* can be detected in breast tissue and milk samples using molecular (i.e., sequencing, PCR) and culture-based methods reported in a previous meta-analysis by Togo et al. [78]. Both *Paracoccus* and *Sphingomonas* can be detected in milk samples using molecular methods [78]. However, *Paracoccus* was not detected in breast milk cultures, while *Sphingomonas* was absent in both milk and breast tissue cultures [78]. Together, the lack of the culture-based detection of *Paracoccus* and *Sphingomonas* in previous breast microbiome studies may indicate their low abundance and stringent culturing requirements, possibility of artefactual detection by a sequencing-based method or a combination of these factors. It is important to highlight the importance of culture-based techniques which add further certainty to indicate the presence of live bacteria within the breast environment.

In addition, the influence of primer selection, study design, and data pre-processing methods on the observed microbial composition can also be a significant confounder [53]. For instance, *Brochothrix*, *Halomonas*, and *Psychrobacter* were identified in some studies and with certain primer sets; however, these were not cultured in either breast tissue or milk [78]. Together, these findings suggest that these bacteria are unlikely to be present in breast tissues.

Microbial diversity and composition were further compared between cancer and other breast tissue types. As reported in previous studies [8,10,15,18,37], alpha diversity was similar between cancer and cancer-adjacent tissues, regardless of the primer sets used in this meta-analysis. In the context of the V3V4 primer set, alpha diversity was higher in normal [33] and mastitis tissues compared to cancer and cancer-adjacent tissues. Additionally, beta diversity was similar between cancer and cancer-adjacent tissues [8,9,10,15,16,18,37], but distinct from normal and mastitis tissues. These findings suggest differences in microbial diversity across tissue types with varying disease phenotypes. Subsequently, we focused on microbial taxa at the genus level between cancer and cancer-adjacent tissues.

As reported in earlier studies [11,15,37], we found that cancer and cancer-adjacent tissues were largely similar (<3 genera significantly different) at the genus level, as demonstrated through both paired and unpaired sample analyses. The only notable differences identified were *Pseudomonas* (elevated in cancer tissues) and *Aliterella* and *Rubrobacter* (decreased in cancer-adjacent tissues) in V4V6 primer set samples. However, *Aliterella* and *Rubrobacter* were not cultured from any breast tissues or milk samples [78], suggesting they may be contaminants or artifacts. Similarly, the TCGA-BRCA microbial dataset showed that cancer and cancer-adjacent tissues were largely similar, with the only differences observed in *Acinetobacter* and *Propionibacterium* in unpaired CLR-abundance analyses. No significant differences were found in the other types of analysis within the TCGA dataset. Together, these findings highlight the similarity between cancer and cancer-adjacent breast tissues, while also underscoring the need for the further validation of datasets using alternative methods.

Lastly, we investigated the relationship between the abundance of *Staphylococcus*, *Acinetobacter*, *Corynebacterium*, and *Streptococcus*, four prominent microbes identified in the TCGA-BRCA dataset, and their association with functional genomic expression signatures and clinical outcomes. While *Acinetobacter* and *Streptococcus* showed no significant associations, *Staphylococcus* was linked to an increased proliferation signature, and *Corynebacterium* was associated with both increased proliferation and a decreased neutrophil signature.

The association between *Staphylococcus* and a proliferation-related signature aligns with previous in vitro studies in lung [86] and oral cancer cells [87]. *Staphylococcus aureus* and its components, such as lipoteichoic acid and peptidoglycan, have been shown to induce proliferation via toll-like receptor-signaling, leading to the activating of downstream pathways such as the NF-κB [86,87,88]. Additionally, *Staphylococcus* superantigens (e.g., staphylococcal protein A, staphylococcal enterotoxins and toxic shock syndrome toxin-1) have been implicated in promoting immune cell proliferation, particularly in T and B cells [89]. However, some studies have reported the anti-proliferative effects of *Staphylococcus* components, including staphopain A and enterotoxin B, in epithelial [90] and glioblastoma cell lines [91]. These discrepancies may stem from variations in experimental models, bacterial strains, bacterial load, or secreted bacterial proteins. Notably, our analysis revealed that patients with a higher *Staphylococcus* abundance in tumor tissue had a shorter overall survival, underscoring its potential clinical significance. Future studies should employ clinically relevant experimental models to further elucidate the role of *Staphylococcus* in breast cancer progression.

We also found that *Corynebacterium* abundance was positively associated with proliferation and negatively associated with neutrophil signatures in breast cancer. While few studies have explored the functional role of *Corynebacterium* in breast cancer, its presence in breast tissues has been well documented [9,10,16,17,25,36,38]. Several reports suggest that *Corynebacterium* is more abundant in healthy breast tissue than in cancerous tissues [10,17]. Given the critical role of neutrophils in shaping breast cancer progression [92], further research is needed to determine whether *Corynebacterium* modulates tumor-associated inflammation in a way that promotes or suppresses disease progression.

Collectively, these findings highlight the potential role of the tumor-associated microbiome, particularly *Staphylococcus* and *Corynebacterium*, in modulating the tumor microenvironment and influencing breast cancer progression. Further studies are warranted to elucidate the mechanistic contributions of these microbes to tumor biology and their potential as therapeutic targets.

This meta-analysis is the first to re-analyze 16S rRNA sequencing data from multiple breast tissue studies using a uniform bioinformatics approach while accounting for batch effects. Despite these efforts, several challenges remain.

Batch-correction tools cannot fully address the variability introduced by differences in DNA extraction protocols, primer sets, or amplified amplicon regions [93,94,95]. Notably, the studies included in our meta-analysis employed diverse extraction methods, which likely influenced the bacterial cell lysis efficiency and DNA recovery [96]. Such methodological variability can lead to a differential representation of microbial taxa, complicating cross-study comparisons and potentially biasing the observed microbial profiles [97]. While our meta-analysis mitigated some of these biases by filtering low-abundance microbes and curating datasets to identify potential contaminants, the variability in DNA extraction protocols, primer sets, and amplified amplicon regions remains a key limitation.

Additionally, the absence of standardized sequencing controls and incomplete metadata in public repositories restricts our ability to effectively control for contamination and background noise. Future studies should adopt standardized approaches to minimize these technical discrepancies and enhance reproducibility.

Molecular methods such as amplicon and metagenomics sequencing, or PCR, are commonly used to identify and profile microbiomes [98]. However, these techniques do not necessarily determine the tissue compartment where the microbes are located, nor do they confirm whether the microbes within tissues are viable [98]. Supplementing sequencing data with culture-based methods can increase confidence in the microbial presence [98]. However, culturing is limited by biases such as selectivity and the inability to grow certain bacteria [98]. For instance, transmission electron microscopy evidence suggests that bacteria in breast tissues may exist intracellularly and lack cell walls [16]. These cell wall-deficient bacteria (L-forms) are particularly challenging to isolate and often require osmoprotective culture media [99].

We also lacked extensive metadata or transcriptomics datasets to comprehensively associate microbial profiles with clinical or tumor phenotypes in the analyzed 16S rRNA datasets. Consequently, these associations rely on the TCGA-BRCA cohort, which included a limited sample size (n = 107).

To better explore host–microbe interactions in breast cancer, we propose integrating multiple approaches. At a single-cell level, methods like INVADEseq (invasion–adhesion-directed expression sequencing) can identify microbes within specific cell subsets and assess their impact on host cell transcriptomes [100,101]. Spatial host-microbiome sequencing [101,102] can further elucidate the spatial distribution of microbial communities within the tumor microenvironment. Combining these techniques with culturomics and transmission electron microscopy will provide a robust framework for understanding the intricate relationships between microbes and breast tissues including breast cancer. However, while these approaches deepen our understanding of microbial presence and associations, most studies to date remain correlative, limiting our ability to establish causality. Functional studies using mouse models [103] have begun to link dysbiosis with breast cancer progression, underscoring the need for experimental validation. Future research integrating spatial and single-cell sequencing with functional assays, such as organoid models, co-culture systems, and gnotobiotic mouse models, will be crucial in determining whether specific microbial taxa actively drive tumor progression or merely reflect disease-associated changes.

## 5. Conclusions

This meta-analysis identified a consensus microbial signature in breast cancer tissues, revealing distinct and consistent microbial profiles shared across multiple studies despite the variability introduced by differences in primer sets and study-specific methodologies. While certain microbes, such as *Staphylococcus* and *Corynebacterium*, show potential associations with tumor progression, further studies are needed to establish their functional roles and clinical relevance. Our findings emphasize the potential of the breast microbiome as a promising target for future therapeutic interventions and diagnostic strategies. However, a key challenge in breast microbiome research remains the variability in study methodologies, which limits reproducibility and cross-study comparisons. Standardized protocols for DNA extraction, sequencing, and data processing are essential for advancing the field. Future studies integrating spatial and functional analyses will be crucial in determining whether these microbial associations hold diagnostic or therapeutic potential in breast cancer.

## Figures and Tables

**Table 1:** The list of studies and tissue samples used for this meta-analysis after pre-processing and filtering.

Study	SRA Project Accession Number	16S rRNA Hypervariable Region	Cancer	Cancer-Adjacent	Normal	Benign	Benign-Adjacent	Mastitis
Kim (2021) [8]	PRJEB37724	V1V3	37	38	0	0	0	0
German (2023) [9]	PRJNA867176	V3V4	31	61	398	0	0	0
Hoskinson (2022) [10]	PRJNA723425	V3V4	42	46	63	0	0	0
Kartti (2023) [11]	PRJNA926328	V3V4	17	14	0	0	0	0
Thyagarajan (2020) [12]	PRJNA637875	V3V4	31	33	0	0	0	0
Zhu (2022) [13]	PRJNA667140	V3V4	0	0	48	0	0	48
Heiken (2016) [45]	PRJNA335375	V3V5	0	16	0		12	0
Li (2022) [19]	PRJNA842933	V4	0	79	0	15	0	0
Esposito (2022) [15]	PRJNA759366	V4V6	34	34	0	0	0	0
Nejman (2020) [16]	PRJNA624822	V6	77	18	0	0	0	0
Urbaniak (2016) [17]	PRJNA323995	V6	0	33	23	0	12	0
Total			269	372	532	15	24	48

**Table 2:** The top phyla and genera in breast tissues using the V3V4 primer set (unrarefied, *n* = 832).

Phylum	Relative Abundance (Mean ± SD)	Prevalence (%)
*Proteobacteria*	42.2 ± 33.1%	83.2%
*Firmicutes*	25.8 ± 28.3%	79.3%
*Actinobacteriota*	25.4 ± 29.7%	73.9%
*Bacteroidota*	3.8 ± 12.7%	35.0%
**Genera**	**Relative Abundance (Mean ± SD)**	**Prevalence (%)**
*Burkholderia-Caballeronia-Paraburkholderia*	11.1 ± 20.6%	50.0%
*Corynebacterium*	9.9 ± 21.1%	41.5%
*Staphylococcus*	9.0 ± 20.3%	42.8%
*Acetobacter*	7.2 ± 16.8%	28.4%
*Ralstonia*	6.8 ± 18.5%	25.5%
*Lactobacillus*	5.1 ± 12.9%	33.7%

**Table 3:** Univariate Cox proportional hazard models for overall survival.

		n	HR (95% CI)	*p*-Value
Age (years)	<65	74		
	≥65	33	2.23 (0.85–5.84)	0.103
*Staphylococcus*	Low	33		
	High	32	4.06 (1.13–14.6)	0.032 *
	Continuous	107	1.40 (1.04–1.88)	0.027 *
*Acinetobacter*	Low	33		
	High	31	0.90 (0.27–2.95)	0.859
	Continuous	107	1.01 (0.83–1.24)	0.891
*Corynebacterium*	Low	34		
	High	31	4.72 (1.02–21.9)	0.048 *
	Continuous	107	1.21 (0.93–1.56)	0.160
*Streptococcus*	Low	32		
	High	31	2.06 (0.41–10.3)	0.236
	Continuous	107	1.20 (0.89–1.63)	0.380

* Denotes *p* < 0.05.

## Data Availability

There is no new sequencing data generated in this study. All data used in this study can be obtained from NCBI SRA.

## Supplementary Materials

The following supporting information can be downloaded at https://www.mdpi.com/article/10.3390/microorganisms13020467/s1; Figure S1: General composition of breast microbiome at the phylum level in 16S rRNA sequenced samples (not rarefied); Figure S2: General composition of breast microbiome at the phylum level in 16S rRNA sequenced samples (rarefied); Figure S3: General composition of breast microbiome at the genus level in 16S rRNA sequenced samples (not rarefied); Figure S4: General composition of breast microbiome at the genus level in 16S rRNA sequenced samples (rarefied); Figure S5: Alpha-diversity index comparison between cancer and cancer-adjacent samples using V1V3 and V4V6 primer sets (not rarefied); Figure S6: Alpha-diversity index comparison between cancer, cancer-adjacent, normal, and mastitis breast tissues samples using V1V3, V3V4, and V4V6 primer sets (rarefied); Figure S7: Beta diversity for cancer and cancer-adjacent samples sequenced using CLR-abundance of V1V3 and V4V6 breast tissue samples; Figure S8: Beta diversity for cancer, cancer-adjacent, normal, and mastitis breast samples sequenced using rarefied relative abundance; Figure S9: Initial TCGA-BRCA analysis without removal of likely contaminants; Figure S10: TCGA-BRCA analysis after removal of likely contaminants; Table S1: Excluded articles that did not meet the eligibility criteria; Table S2: Mean relative abundance of all breast tissue samples (non-rarefied dataset); Table S3: Mean relative abundance of all breast tissue samples (rarefied dataset); Table S4: Prevalence of all breast samples by study at the genera level (not rarefied); Table S5: Prevalence of all breast samples by study at the genera level (rarefied); Table S6: Beta-diversity statistics (PERMANOVA and Beta-dispersion permutation test) based on Euclidean distance of CLR-abundance; Table S7: Beta-diversity statistics (PERMANOVA and Beta-dispersion permutation test) based on Bray–Curtis for rarefied relative abundance dataset; Table S8: Paired sample analyses—Beta-diversity statistics (PERMANOVA and beta-dispersion permutation test) based on Euclidean distance of CLR-abundance (paired); Table S9: Differential abundance analyses at the genus level for V1V3 primer set; Table S10: Differential abundance analyses at the genus level for V3V4 primer set; Table S11: Differential abundance analyses at the genus level for V4V6 primer set; Table S12: Unfiltered genus level relative abundance for all breast tissue from TCGA-BRCA; Table S13: Filtering of TCGA microbes based on 16S rRNA and likely contaminants; Table S14: Mean relative abundance at the genus level for all breast tissue samples for TCGA dataset; Table S15: TCGA beta-diversity statistics (PERMANOVA and Beta-dispersion permutation test); Table S16: Differential abundance analyses at the genus level for TCGA-BRCA; File S1: Search Terms; File S2: Risk of Bias (ROB2); File S3: PRISMA Abstract Check List; File S4: Table of Contents.

## Abbreviations

The following abbreviations are used in this manuscript:

FGES	Functional gene expression signatures
FFPE	Formalin-fixed paraffin-embedded
TCGA	The Cancer Genome Atlas
TCGA-BRCA	The Cancer Genome Atlas Breast Cancer
DADA	Divisive Amplicon Denoising Algorithm
FDR	False discovery rate
ND	Not detected
CLR	Centered log ratio
