# Supplementary material for: A Systematic Review and Meta-Analysis of 16S rRNA and Cancer Microbiome Atlas Datasets to Characterize Microbiota Signatures in Normal Breast, Mastitis, and Breast Cancer
Source: Microorganisms. 2025 Feb 19;13(2):467. doi: 10.3390/microorganisms13020467 (PMC11858161; doi:10.3390/microorganisms13020467)
Supplement: Supplementary file 1 [file microorganisms-13-00467-s001.zip › Figure S1-S10.pdf]

Supplementary Figures

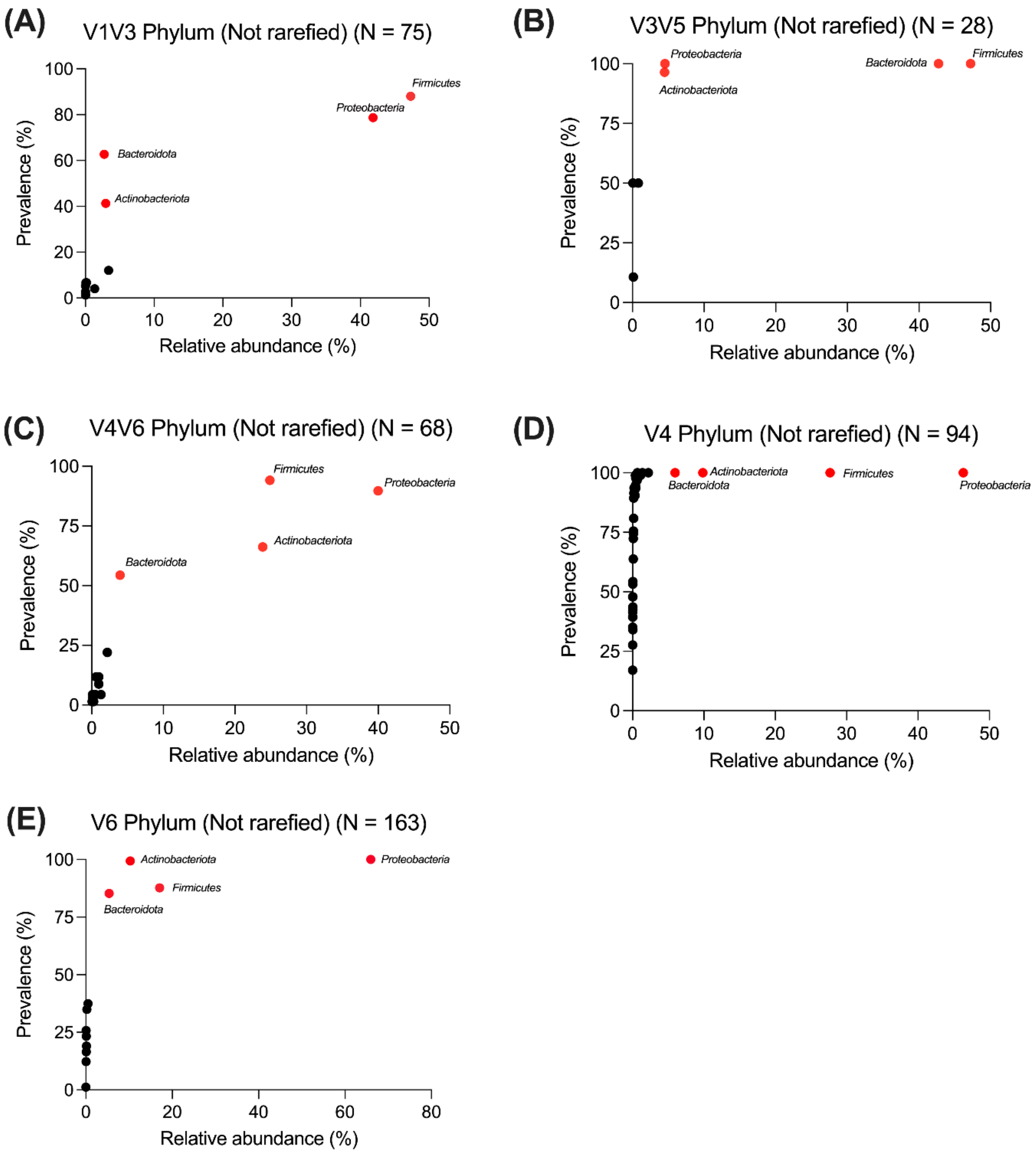

Figure S1: General composition of breast microbiome at the phylum level in 16S rRNA sequenced samples (Not rarefied). Prevalence and mean relative abundance (Not rarefied) at the phylum level for (A) V1V3 (PRJEB37724), (B) V3V5 (PRJNA335375), (C) V4V6 (PRJNA759366), (D) V4 (PRJNA842933), and (E) V6 (PRJNA323995, PRJNA624822) primer sets. Red point highlights top phyla identified.

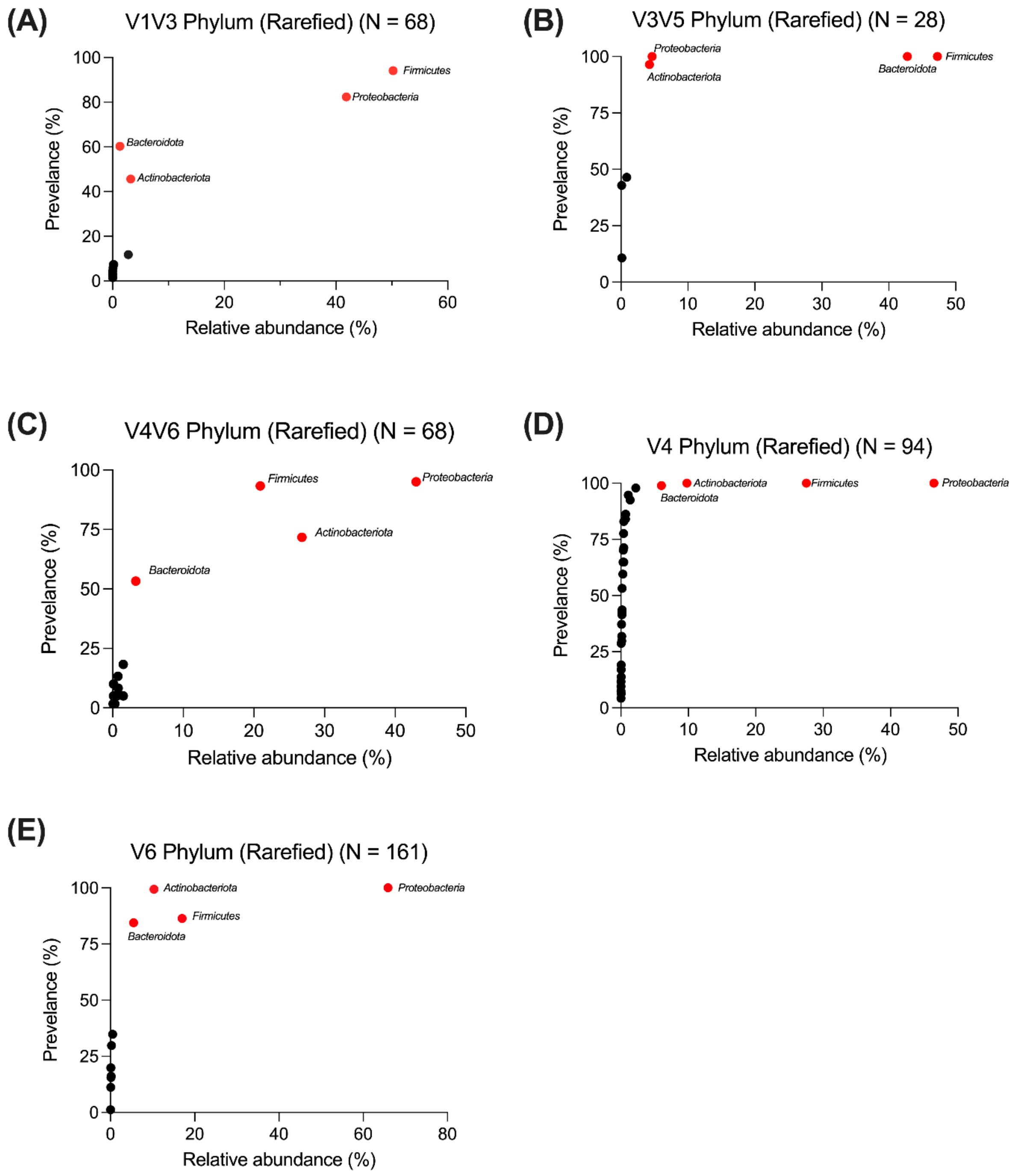

Figure S2: General composition of breast microbiome at the phylum level in 16S rRNA sequenced samples (Rarefied). Prevalence and mean relative abundance (Rarefied) at the phylum level for (A) V1V3 (PRJEB37724), (B) V3V5 (PRJNA335375), (C) V4V6 (PRJNA759366), (D) V4 (PRJNA842933), and (E) V6 (PRJNA323995, PRJNA624822) primer sets. Red point highlights top phyla identified.

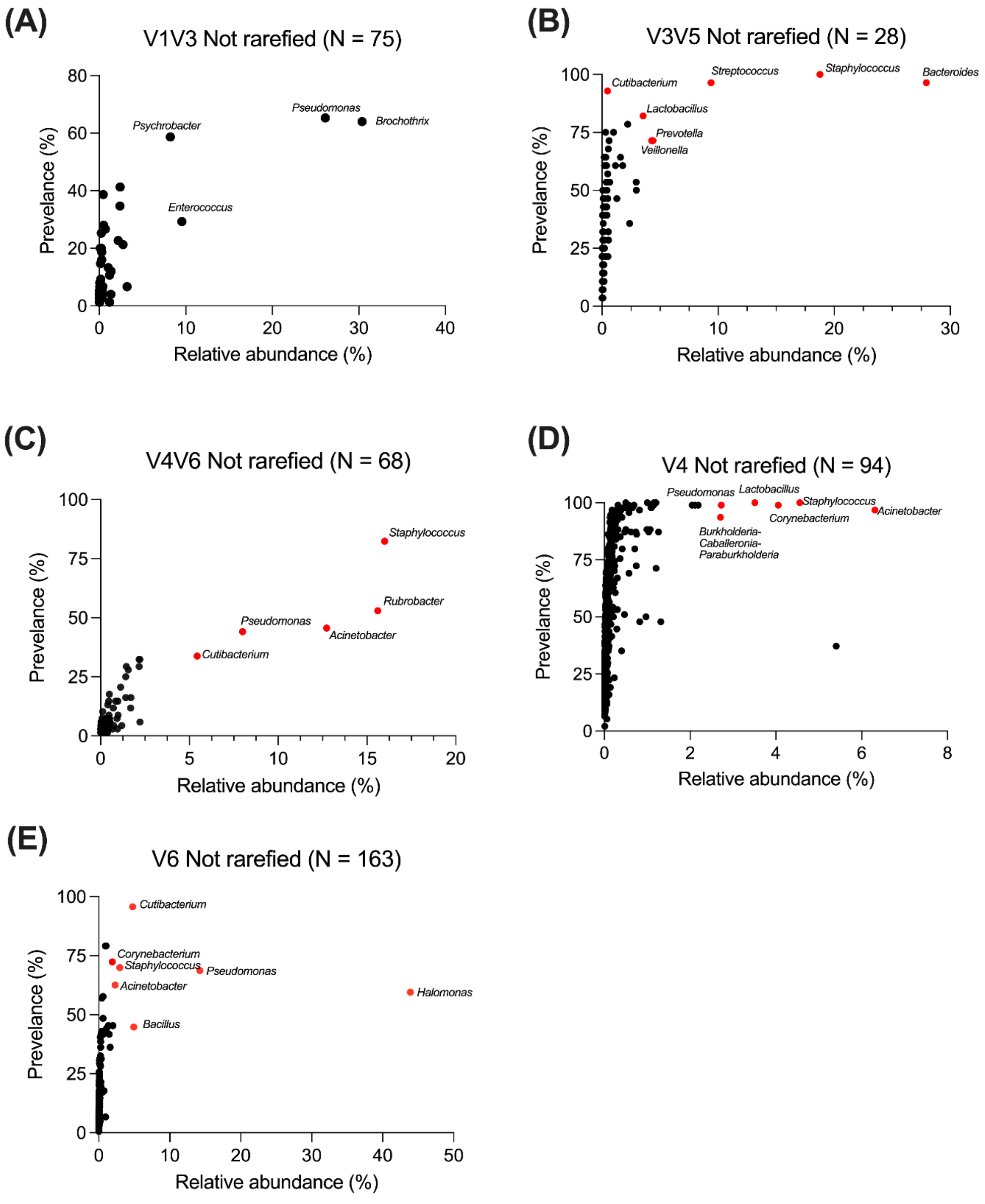

Figure S3: General composition of breast microbiome at the genus level in 16S rRNA sequenced samples (Not rarefied). Prevalence and mean relative abundance (Not rarefied) at the genus level for (A) V1V3 (PRJEB37724), (B) V3V5 (PRJNA335375), (C) V4V6 (PRJNA759366), (D) V4 (PRJNA842933), and (E) V6 (PRJNA323995, PRJNA624822) primer sets. Red point highlights top phyla identified.

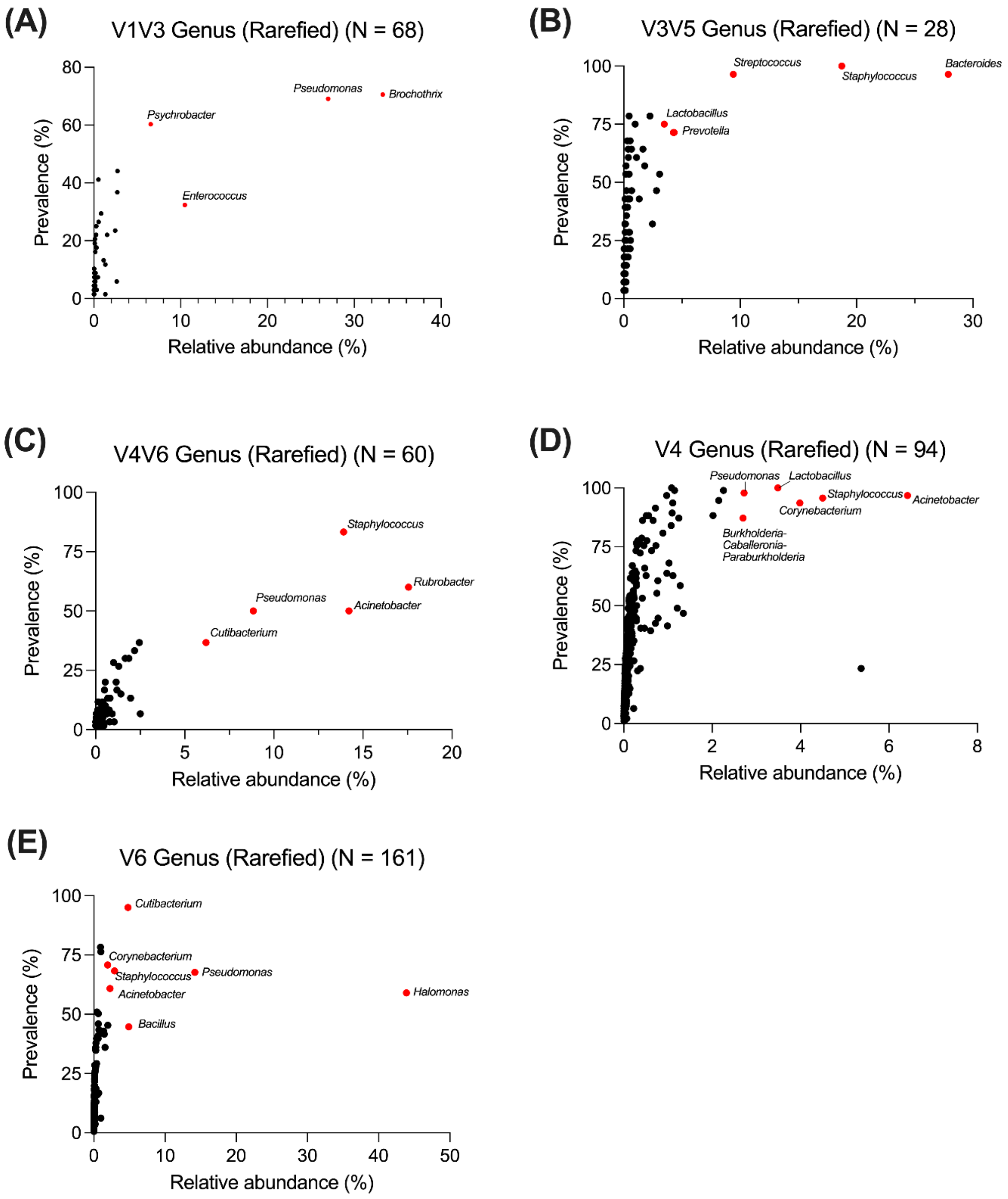

Figure S4: General composition of breast microbiome at the genus level in 16S rRNA sequenced samples (Rarefied). Prevalence and mean relative abundance (Rarefied) at the genus level for (A) V1V3 (PRJEB37724), (B) V3V5 (PRJNA335375), (C) V4V6 (PRJNA759366), (D) V4 (PRJNA842933), and (E) V6 (PRJNA323995, PRJNA624822) primer sets. Red point highlights top phyla identified.

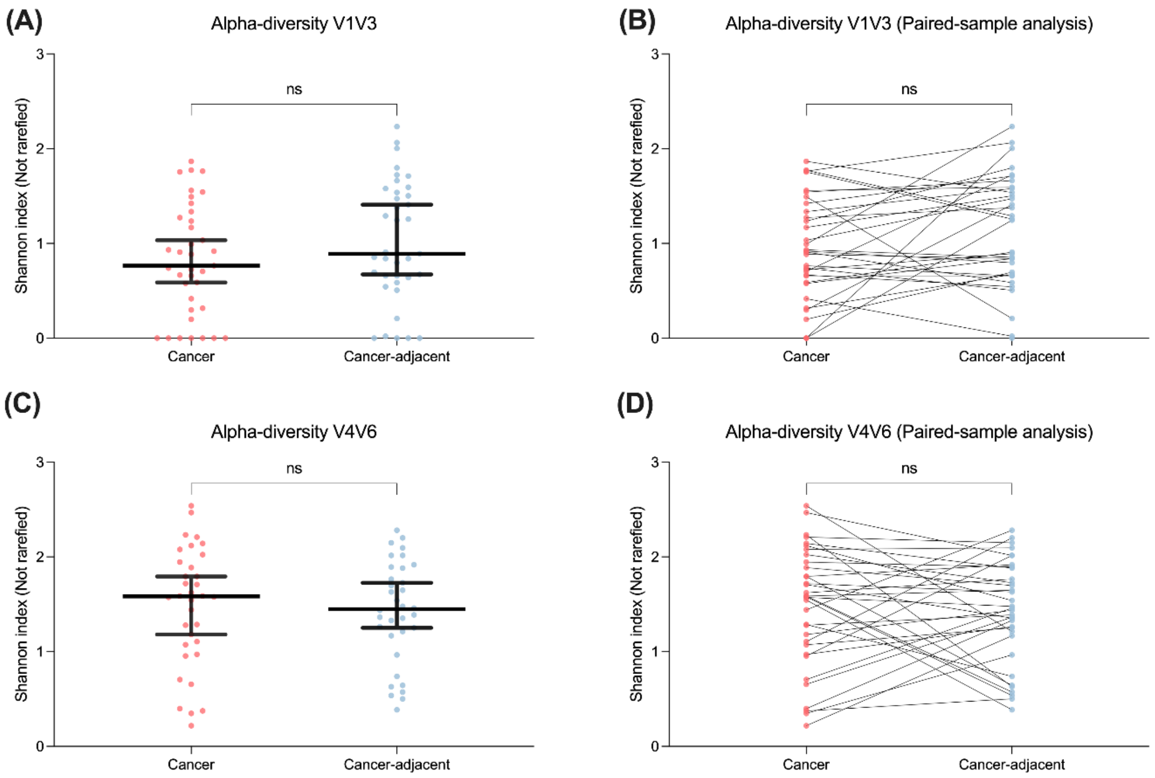

Figure S5: Alpha-diversity index comparison between cancer and cancer-adjacent samples using V1V3 and V4V6 primer sets (Not rarefied). Shannon index (not rarefied) was compared between cancer and cancer-adjacent breast tissues in (A) unpaired V1V3, (B) paired V1V3, (C) unpaired V4V6 and (D) paired V4V6 samples. Mann-Whitney test was used for unpaired analyses, while Wilcoxon test was used for paired analyses. ns – $p > 0.05$.

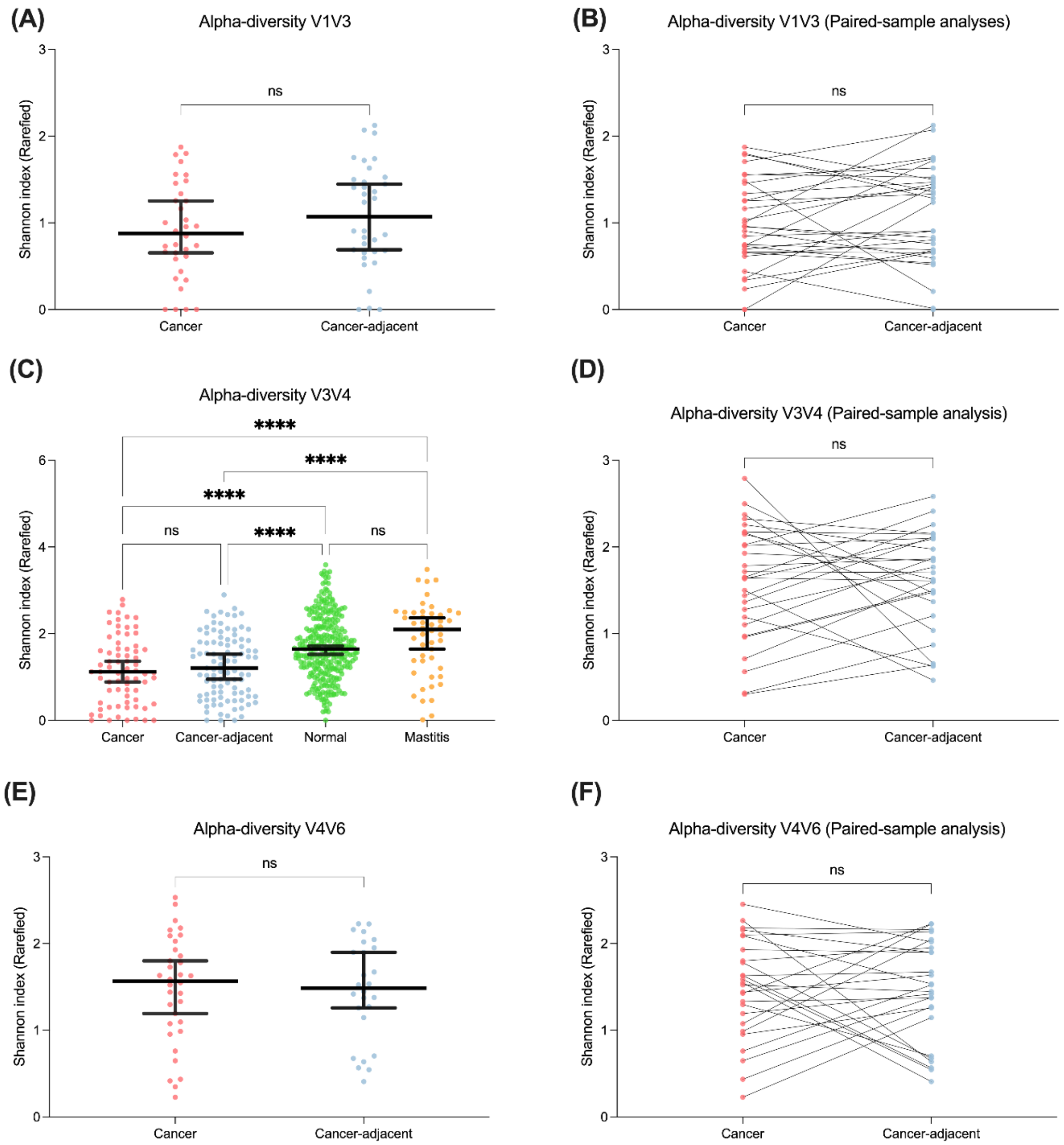

Figure S6: Alpha-diversity index comparison between cancer, cancer-adjacent, normal and mastitis breast tissues samples using V1V3, V3V4, and V4V6 primer sets (Rarefied). Shannon index (Rarefied) was compared between cancer, cancer-adjacent, normal and mastitis breast tissues in (A) unpaired V1V3, (B) paired V1V3, (C) unpaired V3V4, (D) paired V3V4, (E) unpaired V4V6 and (F) paired V4V6 samples. Mann-Whitney test was used for unpaired analyses, while Wilcoxon test was used for paired analyses. For V3V4 primer set, Kruskal-Wallis test with Dunn's multiple comparison was performed for unpaired analyses. ns – $p > 0.05$, ****$p < 0.0001$.

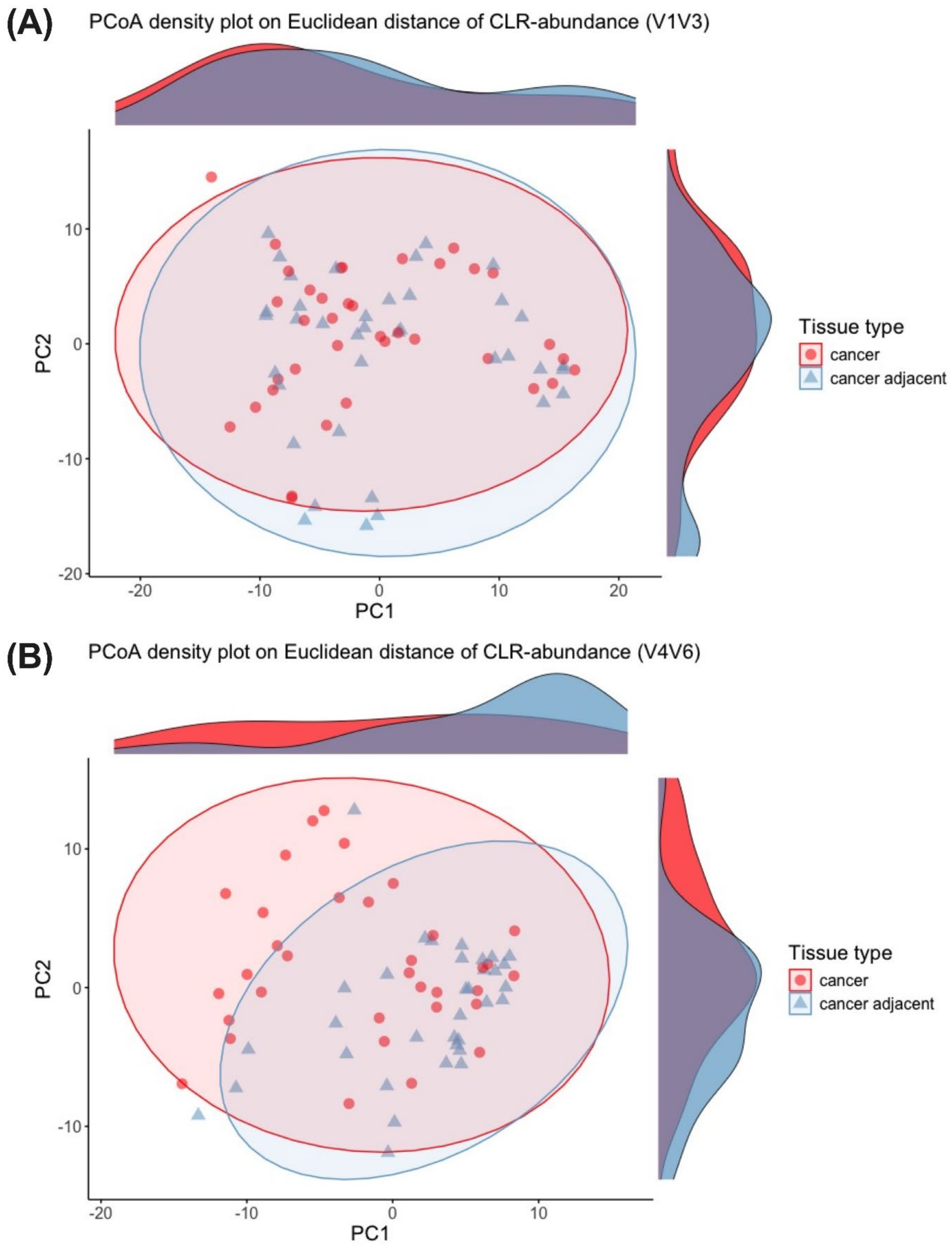

Figure S7: Beta-diversity for cancer and cancer-adjacent samples sequenced using CLR-abundance of V1V3 and V4V6 breast tissue samples. PCoA density plot on Euclidean distance of CLR transformed abundance to represent beta-diversity for (A) V1V3 and (B) V4V6 samples

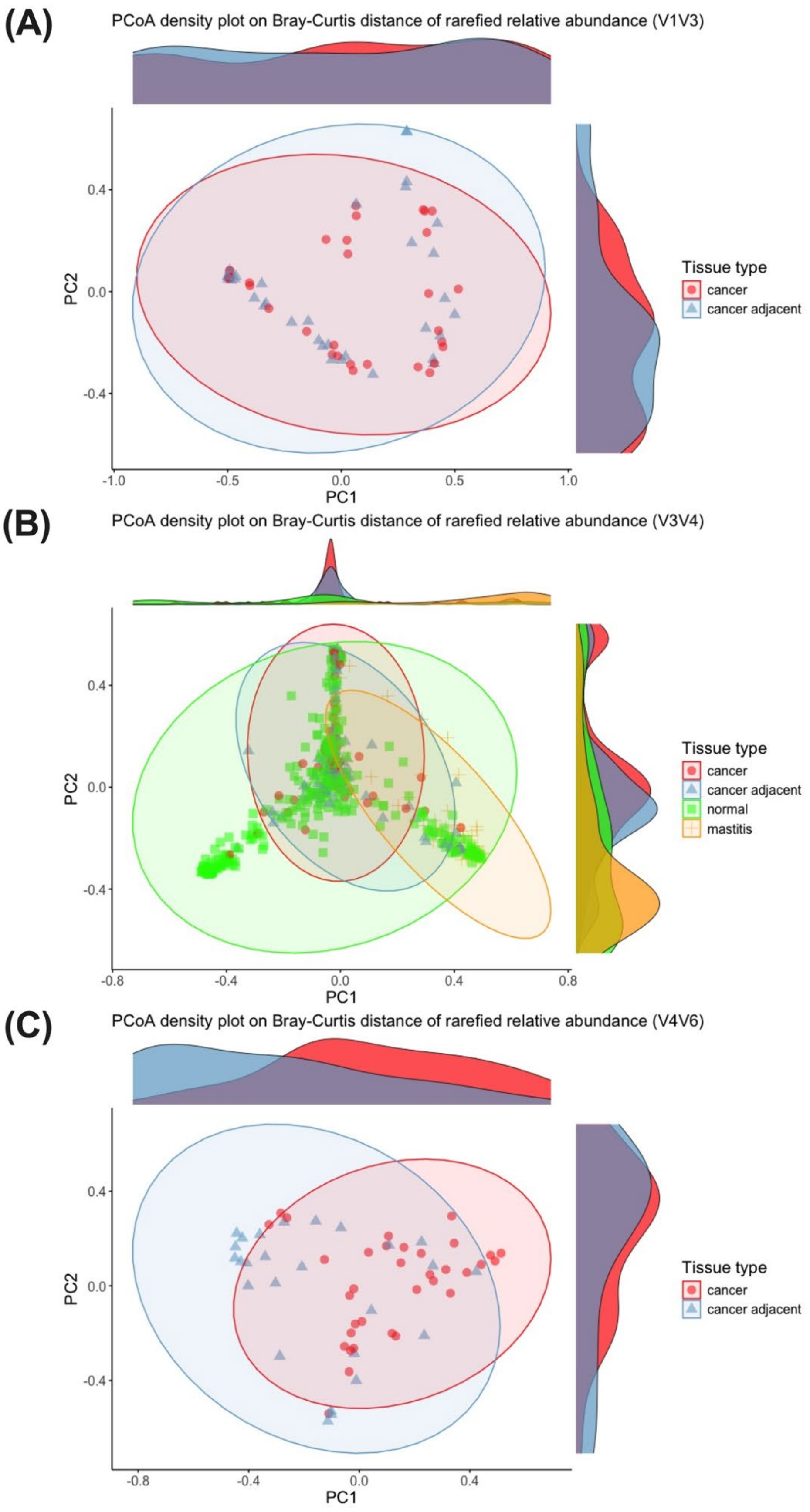

Figure S8: Beta-diversity for cancer, cancer-adjacent, normal and mastitis breast samples sequenced using rarefied relative abundance. PCoA density plot on Bray-Curtis distance of

rarefied relative abundance to represent beta-diversity for (A) V1V3, (B) V3V4, and (C) V4V6 breast tissue samples.

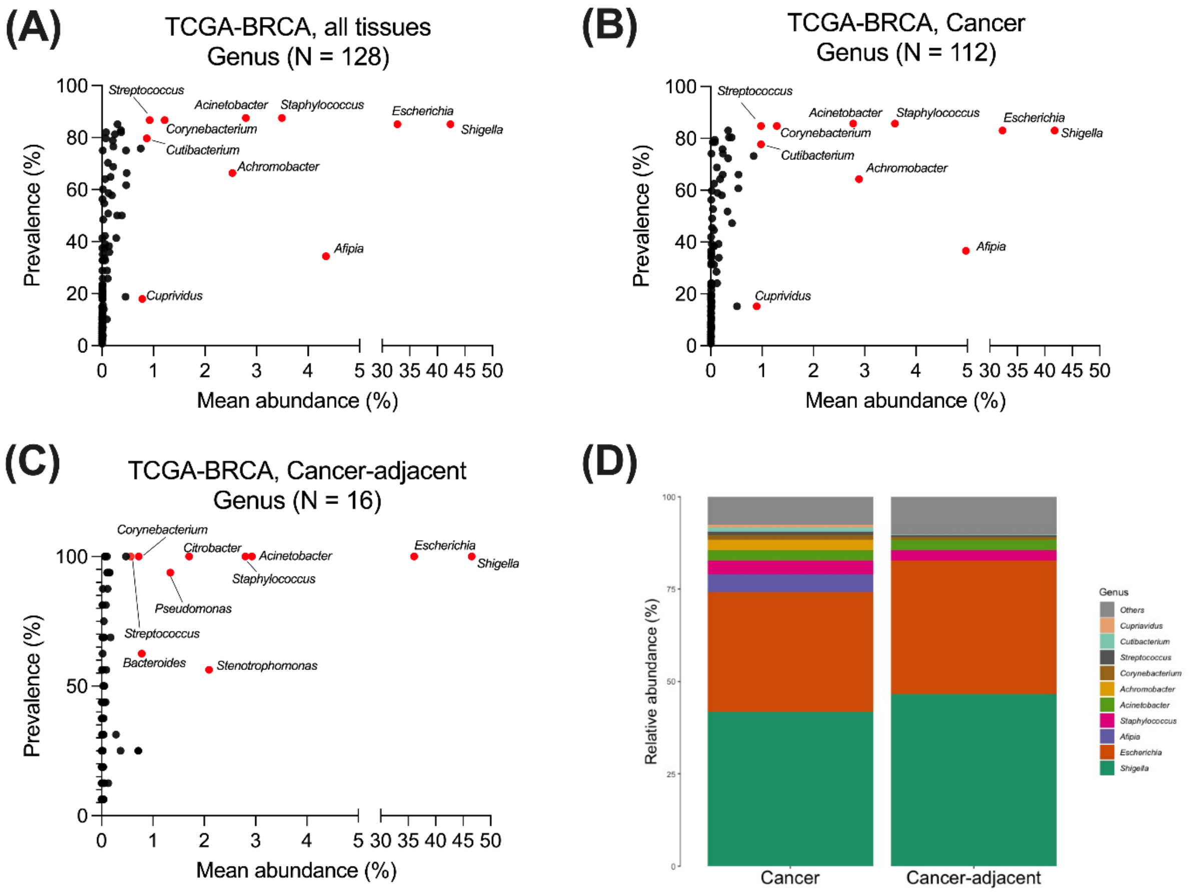

Figure S9: Initial TCGA-BRCA analysis without removal of likely contaminants. Mean relative abundance and prevalence for (A) All tissues, (B) Cancer only, and (C) Cancer-adjacent breast tissues were calculated for TCGA-BRCA cohort using microbial reads generated from Poore et al. (2024). (D) Mean relative abundance of top 10 genera for cancer and cancer-adjacent tissues from TCGA-BRCA.

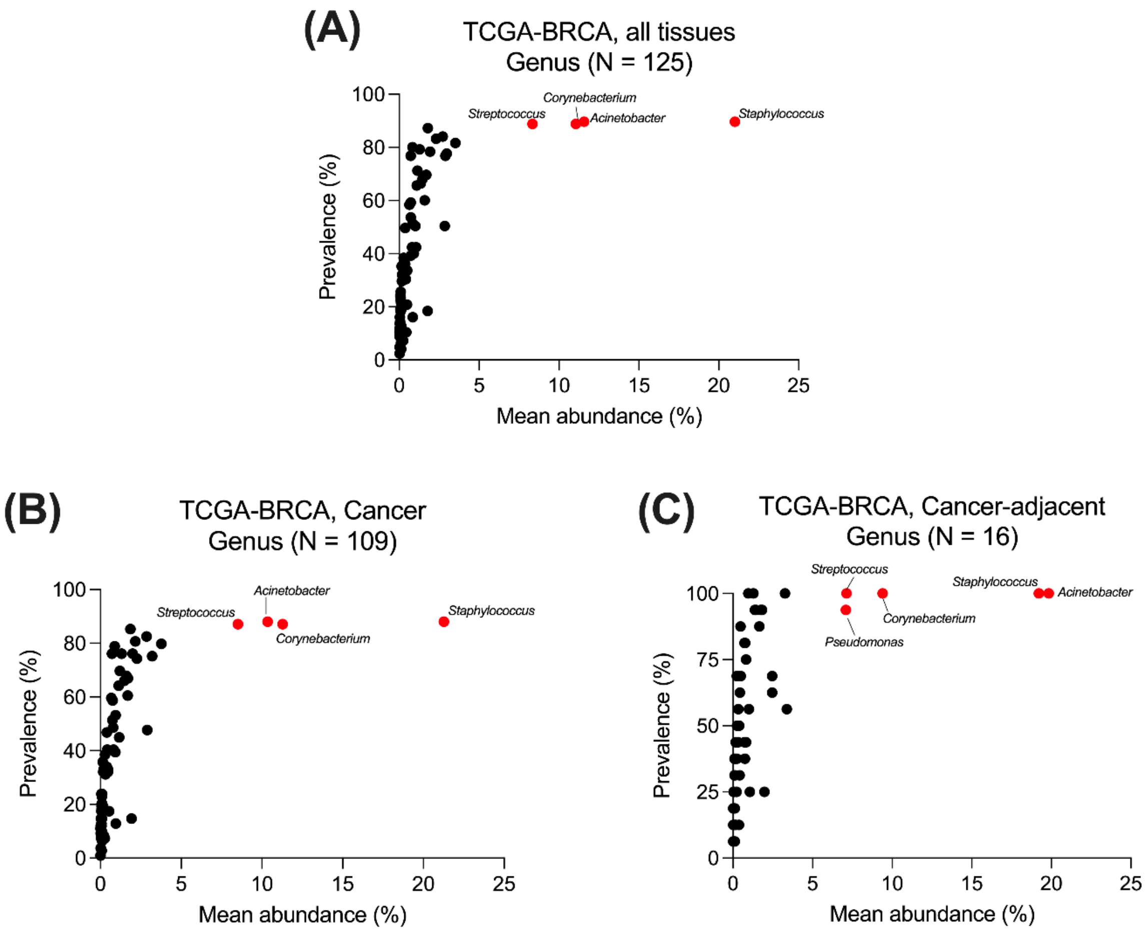

Figure S10: TCGA-BRCA analysis after removal of likely contaminants. Mean relative abundance and prevalence for (A) All tissues, (B) cancer only, and (C) cancer-adjacent breast tissues were calculated for TCGA-BRCA cohort using microbial reads generated from Poore et al. (2024). Genera *Escherichia, Shigella, Afipia* and *Achromobacter* were removed due to unusually high abundance and prevalence as compared to 16S rRNA sequencing dataset. After removal of contaminant reads, three cancer tissues contain 0 microbial reads.
