# Supplementary material for: A Systematic Review and Meta-Analysis of 16S rRNA and Cancer Microbiome Atlas Datasets to Characterize Microbiota Signatures in Normal Breast, Mastitis, and Breast Cancer
Source: Microorganisms. 2025 Feb 19;13(2):467. doi: 10.3390/microorganisms13020467 (PMC11858161; doi:10.3390/microorganisms13020467)
Supplement: Supplementary file 1 [file microorganisms-13-00467-s001.zip › File S1.pdf]

Search terms

PubMed
(breast OR breast tissue OR mammary gland OR breast cancer OR breast tumor OR breast tumour)
AND (microbiome OR microbiota OR microbial OR bacteria OR microbiology)
AND (RNA, Ribosomal, 16S[MeSH] OR 16S OR RNA-seq OR RNA-sequencing OR sequencing OR “microbiota cluster”)
AND English[lang] AND ("1900/01/01"[dp] : "2023/12/31"[dp])
NOT (review[PT] OR "systematic review"[PT] OR "published erratum"[PT] OR letter[PT])
2,821

Scopus
TITLE-ABS-KEY (("breast" OR "breast tissue" OR "mammary gland" OR "breast cancer" OR "breast tumor" OR "breast tumour")
AND ("microbiome" OR "microbiota" OR "microbial" OR "bacteria" OR "microbiology")
AND (“RNA” OR “Ribosomal” OR "16S" OR “RNA-Seq” OR “RNA-sequencing” OR "sequencing" OR "microbiota cluster"))
AND PUBYEAR > 1916 AND PUBYEAR < 2024
AND (LIMIT-TO (DOCTYPE , "ar")) AND (LIMIT-TO (LANGUAGE , "English"))
2,395

EMBASE
("breast" or "breast tissue" or "mammary gland" or "breast cancer" or "breast tumor" or "breast tumour") and ("microbiome" or "microbiota" or "microbial" or "bacteria" or "microbiology") and ("RNA" or "Ribosomal" or "16S" or "RNA-Seq" or "RNA-sequencing" or "sequencing" or "microbiota cluster")

limit 1 to yr="1934 - 2023"
limit 2 to english language
limit 3 to article
1,609
