# Supplementary material for: A Systematic Review and Meta-Analysis of 16S rRNA and Cancer Microbiome Atlas Datasets to Characterize Microbiota Signatures in Normal Breast, Mastitis, and Breast Cancer
Source: Microorganisms. 2025 Feb 19;13(2):467. doi: 10.3390/microorganisms13020467 (PMC11858161; doi:10.3390/microorganisms13020467)
Supplement: Supplementary file 1 [file microorganisms-13-00467-s001.zip › File S4.pdf]

File S4

Table of Contents

A. Overview and description of datasets used in manuscript *2*

B. Batch-correction for tissue samples *2*

B1. Batch-correction for all tissue samples **2**

C. Batch correction for each primer set separately *3*

C1. Batch correction for V3V4 primer set. **3**

C2. Batch correction for V6 primers **6**

Tables: *7*

Table A: Overall and Pairwise PERMANOVA test for all tissue samples (Based on Euclidean distance of CLR-abundance) **7**

Table B: Overall and Pairwise PERMANOVA test for all tissue samples (Based on Bray-Curtis distance of rarefied relative abundance) **10**

Table C: Overall and Pairwise PERMANOVA test for V3V4 primer set samples (Based on Euclidean distance of CLR-abundance) **13**

Table D: Overall and Pairwise PERMANOVA test for V3V4 primer set samples (Based on Bray-Curtis distance of rarefied relative abundance) **14**

A. Overview and description of datasets used in manuscript

This meta-analysis incorporates data from 11 studies, with two studies featuring multiple primer sets while the remaining studies utilised a single primer set (Table 1). The studies with multiple primer sets are PRJNA867176 and PRJNA624822, which included the following primer sets: PRJNA867176 (V1V2, V2V3, V3V4, V4V5, V5V7, V7V9) and PRJNA624822 (V2, V3, V4, V5, V6, V8). Among these, the V3V4 primer set is the most widely represented, with data from four studies comprising 121 cancer samples, 154 cancer-adjacent samples, 509 normal samples, and 48 mastitis samples (Table 1).

Initially, we attempted to include all samples regardless of primer set (Section B1). However, this approach was suboptimal due to substantial batch effects that persisted even after batch correction (Section B1). Consequently, we analysed each primer set separately and applied batch correction for the V3V4 (Section C1) and V6 (Section C2) datasets to improve data consistency.

In our main analyses and supplementary materials, other primer sets from Nejman et al. (2020) and German et al. (2023) were excluded, as these had been comprehensively investigated in prior publications. For the V3V4 primer set, batch correction resulted in slight improvements (Section C1), and the adjusted values were utilised in subsequent analyses. However, for the V6 primer set, no common tissue types were present across studies, leading to an unbalanced design. As a result, batch correction was ineffective, as batch effects could not be separated from biological effects due to the absence of overlapping tissue types between the studies.

Please refer to section B – C for detailed analysis of batch correction.

B. Batch-correction for tissue samples

Batch adjustment was performed by using MMUPHin. Efficacy of batch adjustment was assessed using PERMANOVA on two methods of overall microbial composition (beta-diversity): 1) CLR-normalized abundance (Euclidean distance), 2) Rarefied relative abundance (Bray-Curtis distance).

B1. Batch-correction for all tissue samples

Similar to previous study Yeo et al. (2024), we analyse all breast tissue samples (Tissue type) from different primer sets (Primer) and study batch (BioProject) before and after MMUPHin adjustment. Overall and pairwise PERMANOVA test was performed for all tissues before and after batch adjustment (Refer to Table A and B). Overall, we found significant batch and primer effects were observed ($p < 0.001$) for Euclidean distance of CLR-abundance (Table A) and Bray-Curtis distance of rarefied relative abundance (Table B), even after applying MMUPHin. Notably, while MMUPHin adjustment successfully reduced batch effects in both overall and pairwise PERMANOVA tests, primer effects persisted and, in some cases, exhibited higher R^2 values after batch adjustment (Tables A–B).

Batch-correction tools are generally designed to address technical variations rather than being tailored to specific sources of variability, such as differences in extraction protocols, primers, or amplicon regions amplified. Therefore, to minimise bias introduced by primer set differences, subsequent analyses were conducted separately for each primer set.

C. Batch correction for each primer set separately

In total, there were only two studies employed multiple 16S rRNA primer sets: Nejman et al. (2020) used V2, V3, V5, V6, and V8, while German et al. (2023) used V1V2, V2V3, V3V4, V4V5, V5V7, and V7V9 (Refer to Table 1). All other studies were from a different primer set (Table 1). For consistency and comparability within this meta-analysis, we included only the V6 primer from Nejman et al. (2020) and the V3V4 primer from German et al. (2023), as these were also used in other studies included in the analysis. The other primer sets were excluded as their analyses have been comprehensively explored and reported in published papers. Thus, MMUPHin batch correction was performed on V3V4 and V6 primer sets.

C1. Batch correction for V3V4 primer set.

The V3V4 primer set consists of five studies, containing cancer, cancer-adjacent, normal, and mastitis breast tissues (Table 1). Overall and pairwise PERMANOVA tests were performed for all tissues before and after batch adjustment (Tables C and D). Significant batch effects were observed ($p<0.001$) for both Euclidean distance of CLR-abundance (Table C) and Bray-Curtis distance of rarefied relative abundance (Table D), even after applying MMUPHin. Based on the overall PERMANOVA test for CLR-abundance, the impact of batch adjustment was limited, likely due to large batch effects from normal tissues and similar clustering distances after MMUPHin adjustment (as observed in Figure S11). For Bray-Curtis rarefied relative abundance, the overall PERMANOVA test showed slight improvement (Table D), with batch effects from normal tissues clustering more cohesively after MMUPHin adjustment. Improvements were observed for both CLR-abundance and Bray-Curtis relative abundance in pairwise comparisons involving cancer, mastitis, and cancer-adjacent tissues, where clearer separation was achieved post-adjustment (Figure S11, Table C and D). However, batch effects for normal tissues remained a limiting factor, particularly for CLR-abundance. The V3V4 primer set was used for subsequent analyses in the main manuscript, utilising the batch-adjusted values, as most studies employed this primer set. Data from other primer sets were analysed separately and are presented in the supplementary material.

(A)

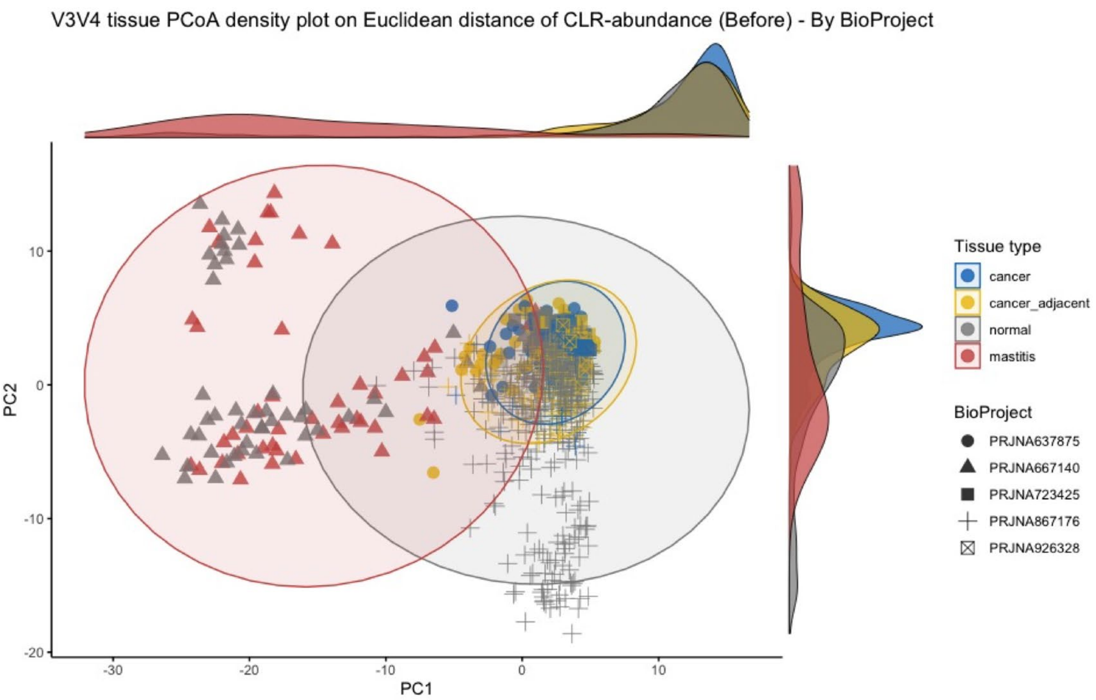

V3V4 tissue PCoA density plot on Euclidean distance of CLR-abundance (Before) - By BioProject
Tissue type
cancer
cancer_adjacent
normal
mastitis
BioProject
PRJNA637875
PRJNA667140
PRJNA723425
PRJNA867176
PRJNA926328
PC1
PC2
10
0
-10
-20
-30

(B)

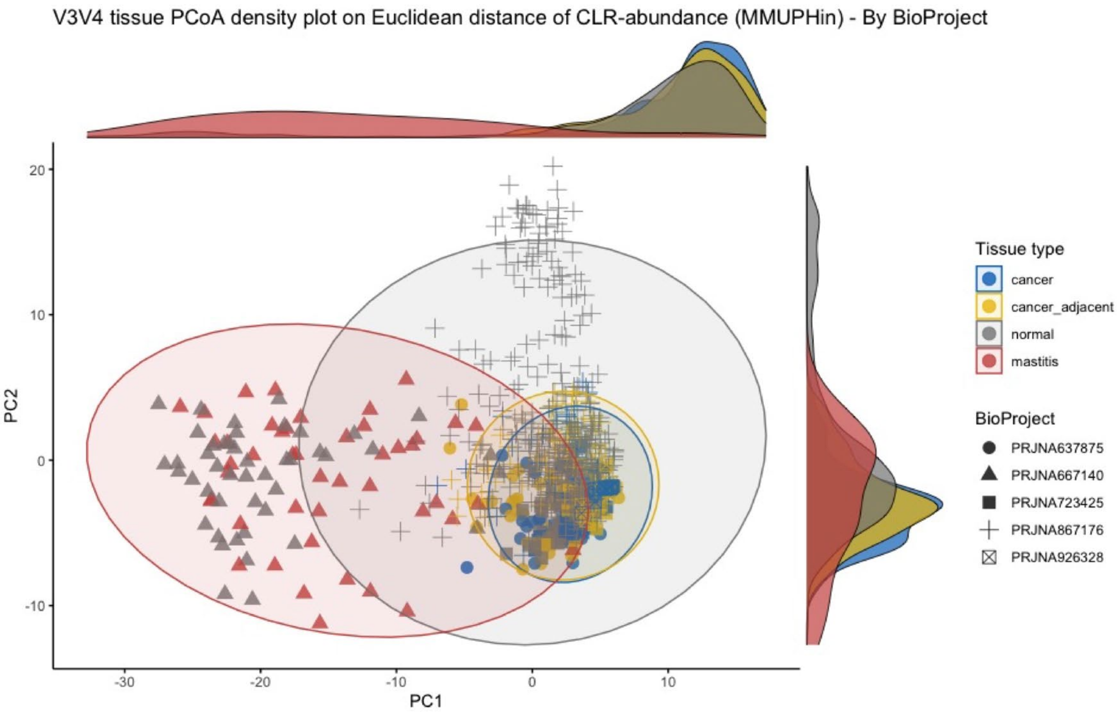

V3V4 tissue PCoA density plot on Euclidean distance of CLR-abundance (MMUPHin) - By BioProject
Tissue type
cancer
cancer_adjacent
normal
mastitis
BioProject
PRJNA637875
PRJNA667140
PRJNA723425
PRJNA867176
PRJNA926328
PC1
PC2
20
10
0
-10
-20
-30

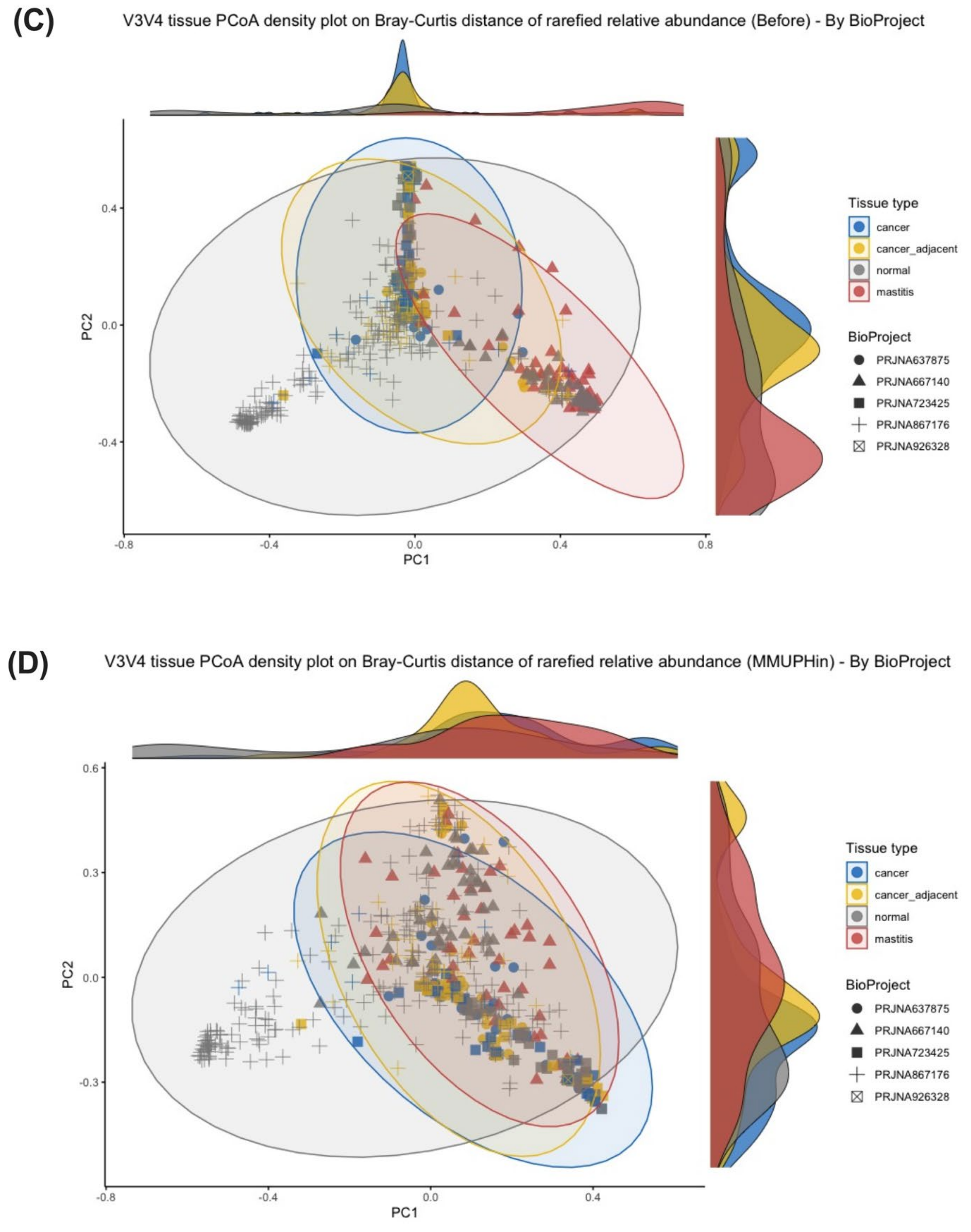

Figure S11: V3V4 PCoA density plots for before and after MMUPHin batch adjustment. PCoA density plot based on Euclidean distance CLR abundance (A) before and (B) after MMUPHin adjustment. PCoA density plot based on Bray-Curtis distance rarefied relative abundance (C) before and (D) after MMUPHin adjustment. For all panels, shape represents the BioProject (study), with ellipse and colour represents the tissue type.

C2. Batch correction for V6 primers

The V6 primer set includes data from two studies: Urbaniak et al. (2016) (PRJNA323995) and Nejman et al. (2020) (PRJNA624822). Nejman et al. (2020) provides data on cancer and cancer-adjacent tissues, while Urbaniak et al. (2016) focuses on benign and benign-adjacent tissues. Due to the unbalanced design, where no "common" tissue types are present across both studies, batch and biological effects could not be disentangled. As a result, unadjusted values were used for analyses of general microbial composition, while for cancer and cancer-adjacent comparisons, only the breast samples from Nejman et al. (2020) were utilised.

Tables:

Table A: Overall and Pairwise PERMANOVA test for all tissue samples (Based on Euclidean distance of CLR-abundance)

	Before		After	
**PERMANOVA (OVERALL)**	**R^2**	**P-value**	**R^2**	**P-value**
Tissue type	0.05236	0.001	0.05101	0.001
BioProject	0.13245	0.001	0.12993	0.001
Primer	0.02476	0.001	0.02546	0.001
**PERMANOVA (Pairwise) Cancer adjacent vs cancer**	**R^2**	**P-value**	**R^2**	**P-value**
Tissue type	0.0196	0.001	0.01998	0.001
BioProject	0.26548	0.001	0.25927	0.001
Primer	0.04181	0.001	0.04331	0.001
**PERMANOVA (Pairwise) Cancer adjacent vs benign adjacent**	**R^2**	**P-value**	**R^2**	**P-value**
Tissue type	0.01064	0.001	0.00948	0.001
BioProject	0.30881	0.001	0.30226	0.001
Primer	0.01818	0.001	0.01926	0.001
**PERMANOVA (Pairwise) Cancer adjacent vs normal**	**R^2**	**P-value**	**R^2**	**P-value**
Tissue type	0.01357	0.001	0.01295	0.001
BioProject	0.12619	0.001	0.12292	0.001
Primer	0.01692	0.001	0.01737	0.001
**PERMANOVA (Pairwise) Cancer adjacent vs benign**	**R^2**	**P-value**	**R^2**	**P-value**
Tissue type	0.03462	0.001	0.03723	0.001
BioProject	0.3035	0.001	0.29597	0.001
Primer	0.01648	0.001	0.01735	0.001
**PERMANOVA (Pairwise) Cancer adjacent vs mastitis**	**R^2**	**P-value**	**R^2**	**P-value**
Tissue type	0.0356	0.001	0.02966	0.001
BioProject	0.28325	0.001	0.27798	0.001
Primer	0.01667	0.001	0.01771	0.001
**PERMANOVA (Pairwise) Cancer vs benign adjacent**	**R^2**	**P-value**	**R^2**	**P-value**
Tissue type	0.01709	0.001	0.01518	0.001
BioProject	0.21013	0.001	0.20749	0.001
Primer	0.0834	0.001	0.08365	0.001

**PERMANOVA (Pairwise) Cancer vs normal**	**R^2**	**P-value**	**R^2**	**P-value**
Tissue type	0.0301	0.001	0.0307	0.001
BioProject	0.08439	0.001	0.0844	0.001
Primer	0.03132	0.001	0.03193	0.001
**PERMANOVA (Pairwise) Cancer vs benign**	**R^2**	**P-value**	**R^2**	**P-value**
Tissue type	0.05885	0.001	0.05932	0.001
BioProject	0.2139	0.001	0.21022	0.001
Primer	0.07158	0.001	0.07174	0.001
**PERMANOVA (Pairwise) Cancer vs mastitis**	**R^2**	**P-value**	**R^2**	**P-value**
Tissue type	0.06182	0.001	0.05168	0.001
BioProject	0.18317	0.001	0.18271	0.001
Primer	0.0727	0.001	0.07366	0.001
**PERMANOVA (Pairwise) Benign adjacent vs normal**	**R^2**	**P-value**	**R^2**	**P-value**
Tissue type	0.00526	0.001	0.00472	0.001
BioProject	0.05057	0.001	0.05008	0.001
Primer	0.01827	0.001	0.01846	0.001
**PERMANOVA (Pairwise) Benign adjacent vs benign**	**R^2**	**P-value**	**R^2**	**P-value**
Tissue type	0.16661	0.001	0.14756	0.001
BioProject	0.23536	0.001	0.22872	0.001
Primer	NA	NA	NA	NA
**PERMANOVA (Pairwise) Benign adjacent vs mastitis**	**R^2**	**P-value**	**R^2**	**P-value**
Tissue type	0.16661	0.001	0.11934	0.001
BioProject	0.23536	0.001	0.88066	0.001
Primer	NA	NA	NA	NA
**PERMANOVA (Pairwise) Normal vs Benign**	**R^2**	**P-value**	**R^2**	**P-value**
Tissue type	0.02075	0.001	0.02183	0.001
BioProject	0.05895	0.001	0.05833	0.001
Primer	0.01741	0.001	0.01755	0.001
**PERMANOVA (Pairwise) Normal vs Mastitis**	**R^2**	**P-value**	**R^2**	**P-value**
Tissue type	0.01892	0.001	0.01602	0.001
BioProject	0.04856	0.001	0.04817	0.001
Primer	0.01755	0.001	0.01776	0.001

**PERMANOVA (Pairwise) Benign vs Mastitis**	**R^2**	**P-value**	**R^2**	**P-value**
Tissue type	0.17848	0.001	0.15624	0.001
BioProject	0.13857	0.001	0.13661	0.001
Primer	NA	NA	NA	NA

Table B: Overall and Pairwise PERMANOVA test for all tissue samples (Based on Bray-Curtis distance of rarefied relative abundance)

	Before		After	
**PERMANOVA (OVERALL)**	**R^2**	**P-value**	**R^2**	**P-value**
Tissue type	0.07822	0.001	0.06909	0.001
BioProject	0.13741	0.001	0.11053	0.001
Primer	0.04672	0.001	0.04604	0.001
**PERMANOVA (Pairwise) Cancer vs Cancer adjacent**	**R^2**	**P-value**	**R^2**	**P-value**
Tissue type	0.03945	0.001	0.04087	0.001
BioProject	0.2707	0.001	0.23709	0.001
Primer	0.09432	0.001	0.09071	0.001
**PERMANOVA (Pairwise) Cancer adjacent vs benign adjacent**	**R^2**	**P-value**	**R^2**	**P-value**
Tissue type	0.0111	0.001	0.00808	0.001
BioProject	0.2882	0.001	0.25181	0.001
Primer	0.03986	0.001	0.0415	0.001
**PERMANOVA (Pairwise) Cancer adjacent vs normal**	**R^2**	**P-value**	**R^2**	**P-value**
Tissue type	0.02156	0.001	0.01939	0.001
BioProject	0.11893	0.001	0.08989	0.001
Primer	0.0245	0.001	0.02557	0.001
**PERMANOVA (Pairwise) Cancer adjacent vs benign**	**R^2**	**P-value**	**R^2**	**P-value**
Tissue type	0.01494	0.001	0.0136	0.001
BioProject	0.29174	0.001	0.25355	0.001
Primer	0.03864	0.001	0.04032	0.001
**PERMANOVA (Pairwise) Cancer adjacent vs mastitis**	**R^2**	**P-value**	**R^2**	**P-value**
Tissue type	0.04058	0.001	0.01979	0.001
BioProject	0.2704	0.001	0.2379	0.001
Primer	0.0374	0.001	0.03921	0.001
**PERMANOVA (Pairwise) Cancer vs benign adjacent**	**R^2**	**P-value**	**R^2**	**P-value**
Tissue type	0.01569	0.001	0.01243	0.001
BioProject	0.27656	0.001	0.24559	0.001
Primer	0.16538	0.001	0.15521	0.001

PERMANOVA (Pairwise) Cancer vs normal	R^2	P-value	R^2	P-value
Tissue type	0.06954	0.001	0.06719	0.001
BioProject	0.10742	0.001	0.08295	0.001
Primer	0.05308	0.001	0.05156	0.001
**PERMANOVA (Pairwise) Cancer vs benign**	**R^2**	**P-value**	**R^2**	**P-value**
Tissue type	0.02463	0.001	0.0207	0.001
BioProject	0.27956	0.001	0.24711	0.001
Primer	0.15891	0.001	0.15001	0.001
**PERMANOVA (Pairwise) Cancer vs mastitis**	**R^2**	**P-value**	**R^2**	**P-value**
Tissue type	0.06221	0.001	0.04018	0.001
BioProject	0.25371	0.001	0.2271	0.001
Primer	0.15172	0.001	0.14353	0.001
**PERMANOVA (Pairwise) Benign adjacent vs normal**	**R^2**	**P-value**	**R^2**	**P-value**
Tissue type	0.0058	0.001	0.00371	0.001
BioProject	0.06206	0.001	0.03651	0.001
Primer	0.02142	0.001	0.02246	0.001
**PERMANOVA (Pairwise) Benign adjacent vs benign**	**R^2**	**P-value**	**R^2**	**P-value**
Tissue type	0.20186	0.001	0.13149	0.001
BioProject	0.23352	0.001	0.17567	0.001
Primer	NA	NA	NA	NA
**PERMANOVA (Pairwise) Benign adjacent vs mastitis**	**R^2**	**P-value**	**R^2**	**P-value**
Tissue type	0.28436	0.001	0.12411	0.001
BioProject	0.71564	0.001	0.87589	0.001
Primer	NA	NA	NA	NA
**PERMANOVA (Pairwise) Normal vs Benign**	**R^2**	**P-value**	**R^2**	**P-value**
Tissue type	0.00902	0.001	0.00782	0.001
BioProject	0.066	0.001	0.03943	0.001
Primer	0.02113	0.001	0.02217	0.001
**PERMANOVA (Pairwise) Normal vs Mastitis**	**R^2**	**P-value**	**R^2**	**P-value**
Tissue type	0.02098	0.001	0.01005	0.001
BioProject	0.06033	0.001	0.03566	0.001
Primer	0.02082	0.001	0.02194	0.001

**PERMANOVA (Pairwise) Benign vs Mastitis**	**R^2**	**P-value**	**R^2**	**P-value**
Tissue type	0.23693	0.001	0.14724	0.001
BioProject	0.12605	0.001	0.08585	0.001
Primer	NA	NA	NA	NA

Table C: Overall and Pairwise PERMANOVA test for V3V4 primer set samples (Based on Euclidean distance of CLR-abundance)

	Before		After	
**PERMANOVA (OVERALL)**	**R^2**	**P-value**	**R^2**	**P-value**
Tissue type	0.06591	0.001	0.0618	0.001
BioProject	0.11949	0.001	0.12006	0.001
**PERMANOVA (Pairwise) Cancer vs Cancer adjacent**	**R^2**	**P-value**	**R^2**	**P-value**
Tissue type	0.00521	0.023	0.00513	0.028
BioProject	0.1379	0.001	0.13487	0.001
**PERMANOVA (Pairwise) Cancer adjacent vs mastitis**	**R^2**	**P-value**	**R^2**	**P-value**
Tissue type	0.16275	0.001	0.15816	0.001
BioProject	0.07418	0.001	0.07093	0.001
**PERMANOVA (Pairwise) Cancer adjacent vs normal**	**R^2**	**P-value**	**R^2**	**P-value**
Tissue type	0.01078	0.001	0.01152	0.001
BioProject	0.14582	0.001	0.14573	0.001
**PERMANOVA (Pairwise) Cancer vs mastitis**	**R^2**	**P-value**	**R^2**	**P-value**
Tissue type	0.19474	0.001	0.18735	0.001
BioProject	0.05264	0.001	0.05124	0.001
**PERMANOVA (Pairwise) Cancer vs normal**	**R^2**	**P-value**	**R^2**	**P-value**
Tissue type	0.01273	0.001	0.0132	0.001
BioProject	0.14461	0.001	0.14503	0.001
**PERMANOVA (Pairwise) Mastitis vs normal**	**R^2**	**P-value**	**R^2**	**P-value**
Tissue type	0.06246	0.001	0.05452	0.001
BioProject	0.11956	0.001	0.12166	0.001

Table D: Overall and Pairwise PERMANOVA test for V3V4 primer set samples (Based on Bray-Curtis distance of rarefied relative abundance)

	Before		After	
**PERMANOVA (OVERALL)**	**R^2**	**P-value**	**R^2**	**P-value**
Tissue type	0.06454	0.001	0.04656	0.001
BioProject	0.15646	0.001	0.10572	0.001
**PERMANOVA (Pairwise) Cancer vs Cancer adjacent**	**R^2**	**P-value**	**R^2**	**P-value**
Tissue type	0.00918	0.023	0.00956	0.033
BioProject	0.16425	0.001	0.147	0.001
**PERMANOVA (Pairwise) Cancer adjacent vs mastitis**	**R^2**	**P-value**	**R^2**	**P-value**
Tissue type	0.11812	0.001	0.06509	0.001
BioProject	0.13546	0.001	0.11997	0.001
**PERMANOVA (Pairwise) Cancer adjacent vs normal**	**R^2**	**P-value**	**R^2**	**P-value**
Tissue type	0.01934	0.001	0.02328	0.001
BioProject	0.18305	0.001	0.11717	0.001
**PERMANOVA (Pairwise) Cancer vs mastitis**	**R^2**	**P-value**	**R^2**	**P-value**
Tissue type	0.15112	0.001	0.07272	0.001
BioProject	0.0928	0.001	0.08629	0.001
**PERMANOVA (Pairwise) Cancer vs normal**	**R^2**	**P-value**	**R^2**	**P-value**
Tissue type	0.02203	0.001	0.02285	0.001
BioProject	0.17874	0.001	0.11206	0.001
**PERMANOVA (Pairwise) Mastitis vs normal**	**R^2**	**P-value**	**R^2**	**P-value**
Tissue type	0.05587	0.001	0.02616	0.001
BioProject	0.17448	0.001	0.10113	0.001
